# Supplementary material for: Identification of a basal system for unwinding a bacterial chromosome origin
Source: EMBO J. 2019 Jun 27;38(15):e101649. doi: 10.15252/embj.2019101649 (PMC6669920; doi:10.15252/embj.2019101649)
Supplement: Supplementary file 1 — Appendix [file EMBJ-38-e101649-s001.pdf]

**APPENDIX - Table of Contents**


**Figure Legends**

Figure Legends S1-S6.

**Supplementary Methods**

Calculated distribution of biotinylated DNA scaffolds on streptavidin coated beads

Strains

Plasmids

Oligonucleotides

**References**

**Figures**

Figures S1-S6.

**Tables**

Table S1-S4.

## Appendix Figure Legends

**Figure S1. DnaA structure and functions. (A)** Primary DnaA domain organization and associated functions. **(B)** Structure of *E. coli* DnaA domain IV bound to a DnaA-box highlighting the helix-turn-helix (HTH) motif and basic loop involved in specific dsDNA binding (PDB ID 1J1V). **(C)** Structure of *A. aeolicus* DnaA domain III filament (monomers are alternately highlighted in blue or green) bound to a single DNA strand (orange) (PDB ID 2HCB).

**Figure S2. Strategy for constructing *incC* and *dnaA* mutants.** Using vectors containing the *B. subtilis incC* region, mutations were introduced by site-directed mutagenesis. Each mutation was verified by sequencing before being subcloned into a targeting vector and again verified by sequencing. Plasmids were transformed into *B. subtilis* and replacement of the endogenous region was confirmed by PCR and sequencing.

**Figure S3. Analysis of DnaA-boxes in *incC.* (A)** An upstream DnaA-box is required for *incC* function. Wild-type (HM1552), *incC*ΔDnaA-box#1/2/3/4 (OH63), *incC*ΔDnaA-box#1/2/3/4/7 (HM1560). **(B)** Neither DnaA-box#6 nor DnaA-box#6/7 are functional *in vivo* when reversed. Wild-type (HM1552), *incC* DnaA-box#6$^{rev}$ (HM1574), *incC* DnaA-box#[6/7]$^{rev}$ (HM1571).

**Figure S4. Overexpression of DnaA is required for origin activity when strains are grown on minimal medium. (A)** The strain used for xylose-dependent overexpression of DnaA. **(B)** Overexpression of DnaA supports the activity of an artificial unwinding region containing only DnaA-box#6/7 *in vivo*. Strains were grown on minimal medium containing glucose and glutamate. *incC* $P_{xyl}$-*dnaA* (HM1854), *incC*$^{art}$DnaA-box#6/7 $P_{xyl}$-*dnaA* (HM1856).

**Figure S5. DnaA$^{CC}$ displays ATP-dependent DNA unwinding activity.** DnaA$^{CC}$ was incubated with a wild-type scaffold in the presence of either ADP or ATP. The two panels are cropped from the same gel.

**Figure S6. DnaA unwinding activity occurs *in cis* and requires ATP and Ile190. (A)** Schematic of an experiment using biotinylated DNA scaffolds captured on streptavidin coated beads. **(B)** DnaA unwinding of DNA scaffolds that are captured on streptavidin coated beads and magnetically immobilized requires ATP and ssDNA binding activity.

**Appendix Supplementary Methods**

*Calculated distribution of biotinylated DNA scaffolds on streptavidin coated beads*

I.

The approximate length of the DNA scaffold is:

(1) $88\ bp \times 0.34\ nm/bp = 30\ nm$

II.

Each sample contained:

(2) 50 µl of beads

The concentration of the beads was:

(3) $\sim 10^7$ beads/µl (Dynabeads MyOne Streptavidin C1, ThermoFisher)

Therefore, the total number of beads used was:

(4) $\sim 50 \times 10^7 = 5\times10^8$ beads

III.

Each sample contained:

(5) 12.5 nM DNA × 54410 Da × 20 µl = 13.6 ng DNA = 0.252 pmol DNA

(6) 0.252 pmol DNA × $6.02\times10^{23}$ DNA molecules/mol = $1.52\times10^{11}$ DNA molecules

Therefore, the number of DNA molecules (Eq 6) per bead (Eq 4) was:

(7) $1.52\times10^{11}$ DNA molecules ÷ $5\times10^8$ beads = 303 DNA molecules/bead

IV.

The approximate surface area [$A=4\pi r^2$] per bead is:

(8) $4 \times 3.14 \times (0.5\times10^3$ nm radius$)^2 = 3.14\times10^6\ nm^2$

The average area of each bead (Eq 8) per DNA molecule (Eq 7) was:

(9) $3.14\times10^6\ nm^2$/bead ÷ 303 DNA molecules/bead = $1.04\times10^4\ nm^2$/DNA molecule

Therefore, the radial distance [$A=\pi r^2$] surrounding each DNA molecule was:

(10) $(1.04\times10^{4}\ nm^{2}/DNA\ molecule \div 3.14)^{1/2} = 57.4\ nm$

V.

Thus, comparing Eq 1 to Eq 10 indicates that, on average, two DNA substrates bound to the same bead are unlikely to come close enough to support an unwinding reaction *in trans*.

VI.

Critically then, the capture of ~300 DNA molecules per bead acts to decrease the effective concentration of substrate in the reaction, which would be predicted to slow the rate of DNA unwinding *in trans*.

*Strains*

Strains are listed in Appendix Table S1. The genotype of all origin mutants was confirmed by DNA sequencing. Transformation of competent *B. subtilis* cells was performed using an optimized two-step starvation procedure as previously described (Anagnostopoulos *et al*, 1961; Hamoen *et al*, 2002). Briefly, recipient strains were grown overnight at 37°C in transformation medium (Spizizen salts supplemented with 1 µg/ml Fe-$NH_4$-citrate, 6 mM $MgSO_4$, 0.5% glucose, 0.02 mg/ml tryptophan and 0.02% casein hydrolysate) supplemented with IPTG or xylose where required. Overnight cultures were diluted 1:17 into fresh transformation medium supplemented with IPTG or xylose where required and grown at 37°C for 3 hours with continual shaking. An equal volume of prewarmed starvation medium (Spizizen salts supplemented with 6 mM $MgSO_4$ and 0.5% glucose) was added and the culture was incubated at 37°C for 2 hours with continual shaking. DNA was added to 300 µl cells and the mixture was incubated at 37°C for 1 hour with continual shaking. 20-200 µl of each transformation was plated onto selective media supplemented with IPTG or xylose where required and incubated at 37°C for 24-48 hours.

TR671 [*trpC2 aprE*::*kan*(*lacI* $P_{spac}$-*repN/oriN*) *amyE*::*spc*($P_{xyl}$-*dnaA*)] was constructed by transformation with a PCR product generated by three-way Gibson assembly (NEBuilder HiFi). The *dnaA* gene with its native leader sequence was amplified with oTR1858 (5´-AAATGCATCTTTTAATGTGTACGAATGGTAAGCGCCATTTG-3´) and oTR1866 (5´-CACGCAGAATCTATTTAAGCTGTTCTTTAATTTCTTTTACATGCTG-3´) using 168CA genomic DNA as template. The flanking region containing *amyE'-spc*-$P_{xyl}$ was amplified using oTR95 (5´-ACTCAAGACGATAGTTACCGGATAAG-3´) and oTR1857 (5´-ACACATTAAAAGATGCATTTTATGTCATATTGTAAGTAAGTTGC-3´) using pSG1728 as template (Lewis *et al*, 1999). The flanking region containing '*amyE* was amplified using oTR1867 (5´-GCTTAAATAGATTCTGCGTGACATCCCATCGATC-3´) and oTR406 (5´-GTCTGACGCTCAGTGGAAC-3´) using pSG1728 as template.

TR672 [*trpC2 aprE*::*kan*(*lacI* $P_{spac}$-*repN/oriN*) *amyE*::*spc*(*xylR* $P_{xyl}$-*dnaA-dnaN*)] was constructed by transformation with a PCR product generated by three-way Gibson assembly (NEBuilder HiFi). The *dnaA-dnaN* operon with its native leader sequence was amplified with oTR1876 (5´-ACATAAAATGCATCTTTTAATGTGTACGAATGGTAAGCGCCATTTG-3´) and oTR1877 (5´-CGGCGCTCAGGATCCTTAATAGGTTCTGACAGGAAGGATAAGCTGTAC-3´) using TR215 genomic DNA as template. The flanking region containing *amyE'-spc-xylR*-$P_{xyl}$ was amplified using oTR1874 (5´-TGAAGGTCGCGCGCATTCC-3´) and oTR1875 (5´-TTCGTACACATTAAAAGATGCATTTTATGTCATATTGTAAGTAAGTTGCACATTAG-3´) using pHM610 as template. The flanking region containing '*amyE* was amplified using oTR1878 (5´-GTCAGAACCTATTAAGGATCCTGAGCGCCGGTCG-3´) and oTR1879 (5´-GCATCGCCGGCATGTCCCC-3´) using pHM610 as template.

*Plasmids*

Plasmids are listed in the Appendix Table S2 and all mutations were confirmed by DNA sequencing (sequences are available upon request). DH5α ($F^-$ Φ80*lac*ZΔM15 Δ(*lac*ZYA-*arg*F) U169 *rec*A1 *end*A1 *hsd*R17($r_k^-$, $m_k^+$) *pho*A *sup*E44 *thi*-1 *gyr*A96 *rel*A1 $\lambda^-$) was used for plasmid construction. Descriptions, where necessary, are provided below. FastCloning (Li *et al*, 2011) was used with minor modifications. PCR products (15 µl from a 50 µl reaction) were mixed and then subjected to a heating/cooling regime: two cycles of 98°C for 2 minutes → 25°C for 2 minutes, then one cycle of 98°C for 2 minutes → 25°C for 60 minutes. After cooling DpnI restriction enzyme (1 µl) was added to digest parental plasmids and the mixtures were incubated at 37°C for ~4 hours. Following digestion 10 µl of the PCR mixture was transformed into chemically competent *E. coli* DH5α.

pHM293 [*bla phleo*] was generated by ligation of a BamHI fragment containing *phleo*$^R$ from pIC22 (Steinmetz *et al*, 1994) with pLOSS (Claessen *et al*, 2008) cut with BglII.

pHM294 [*bla phleo* P$_{xylR}$-*xylR*] was generated by ligation of a BlnI-NotI PCR product containing P$_{xylR}$-*xylR* (5′-TTCGCAAGAAGCGGCCGCATGAGATTGAGCCATGTGATTTCC-3′ + 5′-GGATCCCTAGGAATTACATTGTAATCATGTCCAG-3′ and HM715 genomic DNA as template) with pHM293 cut with BlnI-NotI.

pHM367 [*bla 'dnaA incC dnaN'*] was generated by ligation of a 1382 base pair EcoRV fragment from pHM327 with pUC18 (Norrander *et al*, 1983) cut with HincII.

pHM510 [*bla aprE' kan lacI* P$_{spac}$-P$_{xylR}$-*xylR 'aprE*] was generated by ligation of an Asp718I-XbaI PCR product containing P$_{xylR}$-*xylR* from pHM294 into pHM446 (Richardson *et al*, 2016) cut with Asp718I-XbaI.

pHM610 [*bla amyE'* P$_{xyl}$-*dnaAchi xylR spc 'amyE*] was generated by FastCloning. The plasmid backbone was amplified with oHM534 (5´-CTATTGGATCAATGACTTTCGGTTTTGAGGAAGG-3´) + oHM535 (5´-GAGCGGTTAAGGATCCTGAGCGCCGGTC-3´) using pKM322 [*bla amyE'* P$_{xyl}$-*dnaA-gfp xylR spc 'amyE*] (Wagner *et al*, 2009) as template. Domain IV of *T. maritima* was amplified with oHM532 (5´-GAAAGTCATTGATCCAATAGATGAACTCATAGAGATC-3´) + oHM533 (5´-CTCAGGATCCTTAACCGCTCAGGGCTCTC-3´) using pTHMA-1 [*bla araC* P$_{BAD}$-*dnaA*$^{Tm}$] (Noguchi *et al*, 2015) as template.

pTR207 [*bla 'dnaA incC*$^{art}$(DnaA-box#6/7) *dnaN'*] was generated by FastCloning. The plasmid backbone was amplified with oTR382 (5´-CCAATACGCACTATTTAAGCTGTTCTTTAATTTCTTTTACATGCTG-3´) + oTR383 (5´-ATATAATTATCATTATCCGTTAGGAGGATAAAAATGAAATTCAC-3´) using pHM367 as template. Artificial *incC* was amplified with oTR380 (5´-GCTTAAATAGTGCGTATTGGGCGCTCTTC-3´) + oTR381 (5´-

ACGGATAATGTATTAATATATATATTTATAAAAAATAGTAGAAGTAATAGTAGGGCCTG-3´) using pTR204 as template.

<u>pTR424</u> [*bla 'dnaA incC*$^{art}$(DnaA-box#/6/7) *dnaN'* ΔDnaA-box(nt866-874) ΔDnaA-box(nt772-780) Δnt1654-1754] was generated to delete the two DnaA-boxes present within pUC18 and to delete the sequence homologous to *incC*$^{art}$. For clarity, only the relevant *incC*$^{art}$ genotype of pTR424 derivatives is annotated in the Plasmid list.

<u>pTR606</u> [*bla 'dnaA incC*$^{art}$(DnaA-box#CR$^{44}$/6/7) *dnaN'* Δnt3431-250] was generated to delete the sequence homologous to *incC*$^{x_art}$. For clarity, only the relevant *incC*$^{x_art}$ genotype of pTR606 derivatives is annotated in the Plasmid list.

<u>pTR608</u> [*bla 'dnaA incC*$^{x\_art}$(DnaA-box#CR$^{44}$/6/7) *dnaN'*] was generated by FastCloning. The plasmid backbone was amplified with oTR991 (5´-TCTAAATACACTATTTAAGCTGTTCTTTAATTTCTTTTAC-3´) + oTR992 (5´-CATTCGCCATTGCGTATTGGGCGCTCTTC-3´) using pTR606 as template. Extended artificial *incC* was amplified with oTR993 (5´-GCTTAAATAGTGTATTTAGAAAAATAAACAAATAGGGGTTCCG-3´) + oTR994 (5´-CCAATACGCAATGGCGAATGGCGCCTGATG-3´) using pUC18 as template.

<u>pTR615</u> [*bla 'dnaA incC*$^{art}$(DnaA-box#/6/7) *dnaN'* Δnt1615-1738] was generated to delete the sequence homologous to *incC*$^{art}$ from the plasmid backbone. For clarity only the relevant *incC*$^{art}$ genotype of pTR615 derivatives is annotated in the Plasmid list.

<u>pTR634</u> [*bla bla 'dnaA incC dnaN cat recF* Δnt5617-6160] was generated to delete the sequence homologous to *incC*$^{x\_art}$.

*Oligonucleotides*

All oligonucleotides were purchased from Eurogentec. Oligonucleotides used for plasmid construction are listed in Appendix Table S3. Oligonucleotides used to construct DNA scaffolds were purified by reverse phase HPLC and are listed in Appendix Table S4.

## APPENDIX REFERENCES

Anagnostopoulos C, Spizizen J (1961) Requirements for transformation in *Bacillus subtilis*. ***J Bacteriol*** **81**: 741-6

Claessen D, Emmins R, Hamoen LW, Daniel RA, Errington J, Edwards DH (2008) Control of the cell elongation-division cycle by shuttling of PBP1 protein in *Bacillus subtilis*. ***Mol Microbiol*** **68**: 1029-46

Hamoen LW, Smits WK, de Jong A, Holsappel S, Kuipers OP (2002) Improving the predictive value of the competence transcription factor (ComK) binding site in *Bacillus subtilis* using a genomic approach. ***Nucleic Acids Res*** **30**: 5517-5528

Lewis PJ, Marston AL (1999) GFP vectors for controlled expression and dual labelling of protein fusions in *Bacillus subtilis*. ***Gene*** **227**: 101-109

Li C, Wen A, Shen B, Lu J, Huang Y, Chang Y (2011) FastCloning: a highly simplified, purification-free, sequence- and ligation-independent PCR cloning method. ***BMC Biotechnol*** **11**: 92

Noguchi Y, Sakiyama Y, Kawakami H, Katayama T (2015) The Arg fingers of key DnaA protomers are oriented inward within the replication origin *oriC* and stimulate DnaA subcomplexes in the initiation complex. ***J Biol Chem*** **290**: 20295-312

Norrander J, Kempe T, Messing J (1983) Construction of improved M13 vectors using oligodeoxynucleotide-directed mutagenesis. ***Gene*** **26**: 101-6

Richardson TT, Harran O, Murray H (2016) The bacterial DnaA-trio replication origin element specifies single-stranded DNA initiator binding. ***Nature*** **534**: 412-6

Steinmetz M, Richter R (1994) Plasmids designed to alter the antibiotic resistance expressed by insertion mutations in *Bacillus subtilis*, through in vivo recombination. ***Gene*** **142**: 79-83

Wagner JK, Marquis KA, Rudner DZ (2009) SirA enforces diploidy by inhibiting the replication initiator DnaA during spore formation in *Bacillus subtilis*. ***Mol Microbiol*** **73**: 963-74

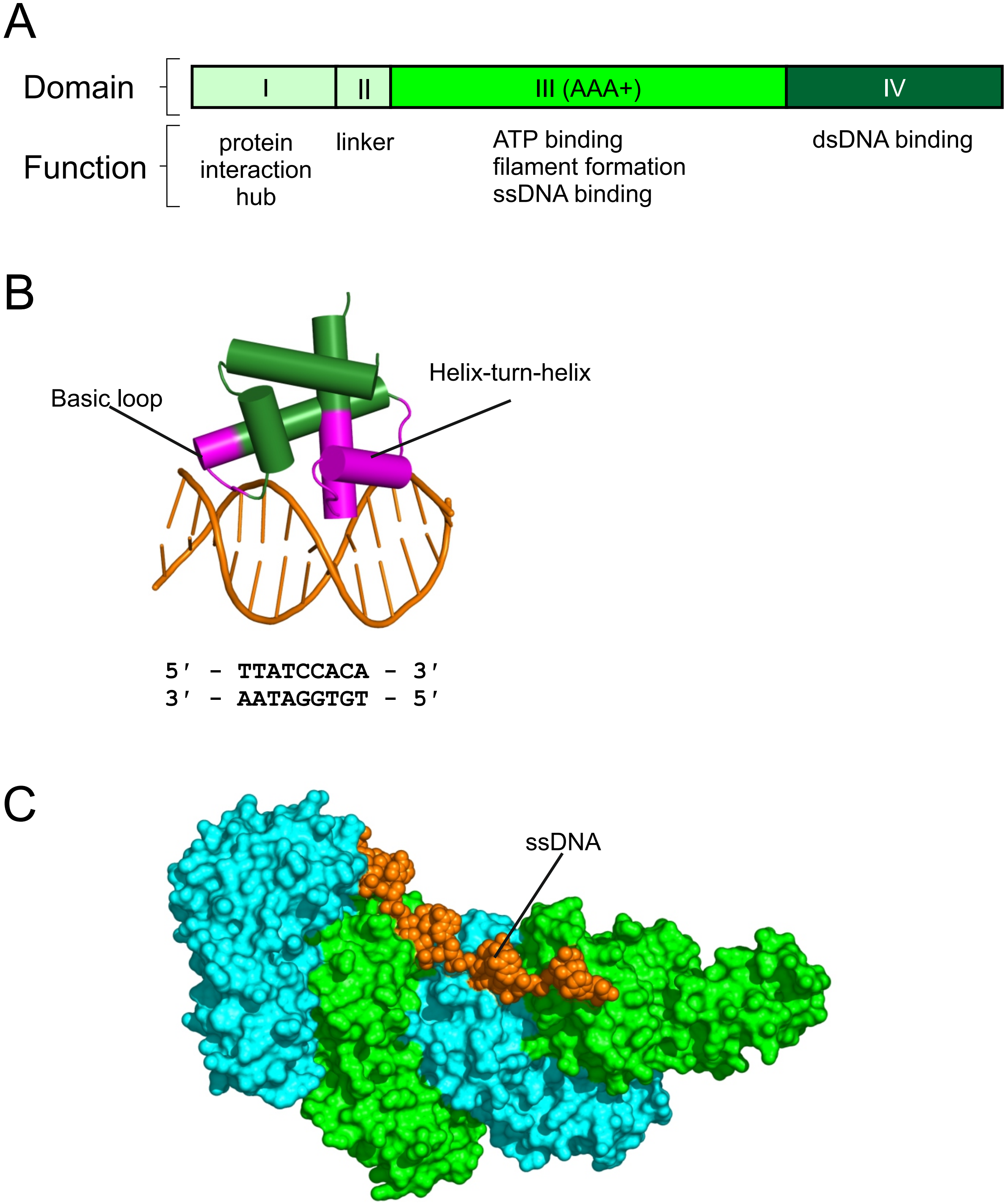


Appendix Figure S1

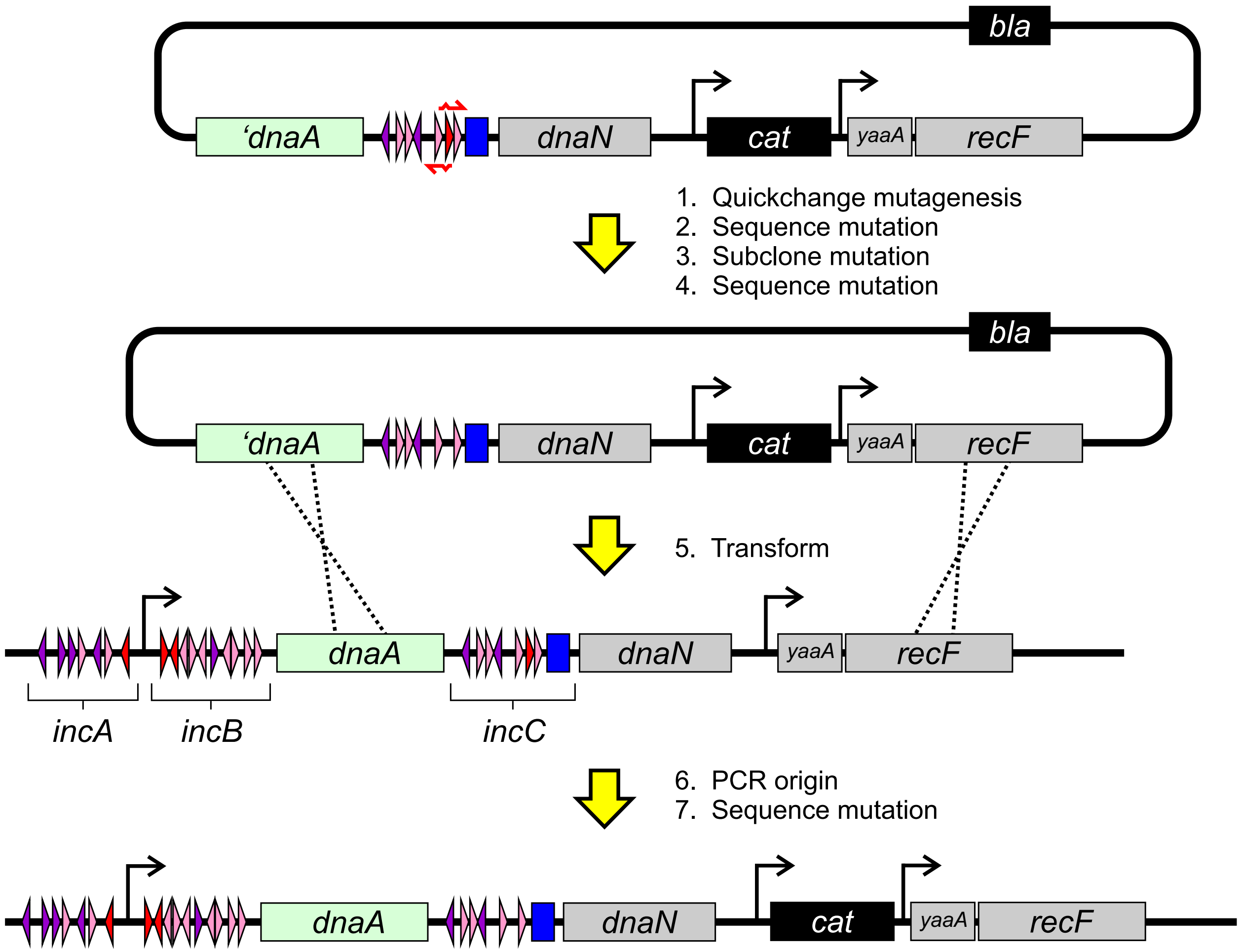


Appendix Figure S2

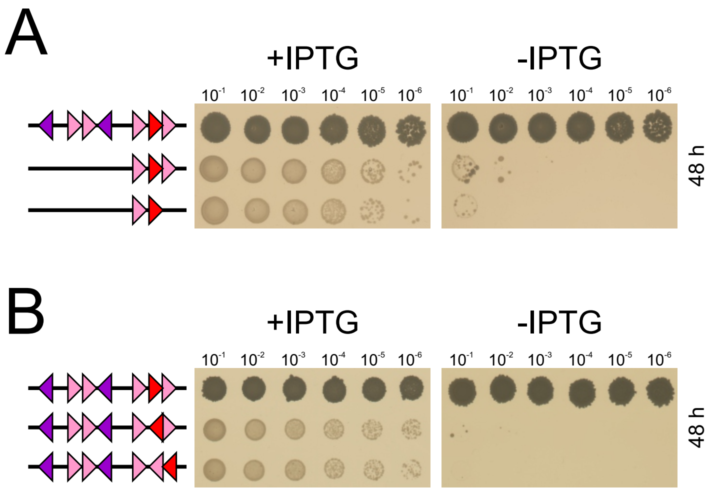


Appendix Figure S3

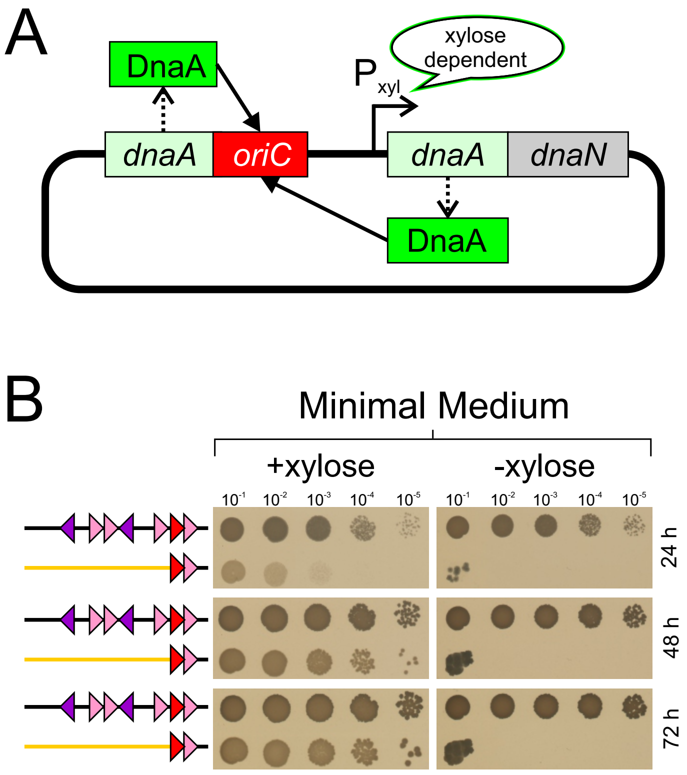


Appendix Figure S4

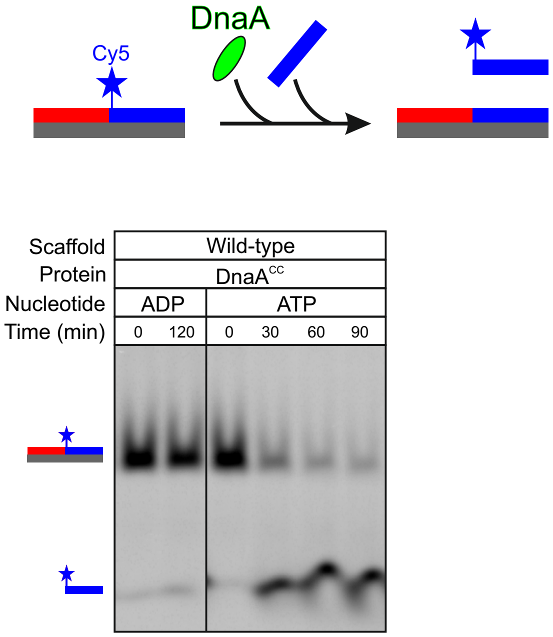


Appendix Figure S5

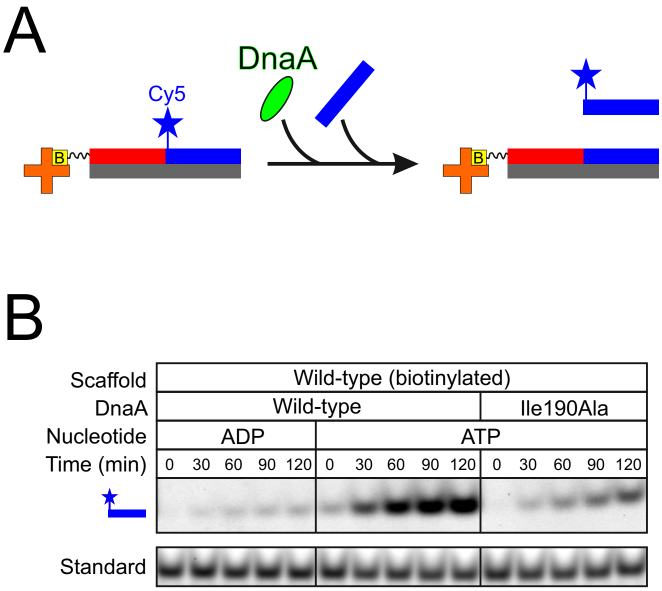


Appendix Figure S6

# Appendix Table 1. Strains

| Strain | Genotype | Parent strain | DNA used for construction | Reference |
|---|---|---|---|---|
| DS6 | trpC2 aprE ::kan(lacI $P_{spac}$-repN/oriN) dnaA $^{E183A}$ ::cat | HM1108 | pDS22 | This work |
| DS18 | trpC2 aprE ::kan(lacI $P_{spac}$-repN/oriN) dnaA $^{I190A}$ ::cat | HM1108 | pDS50 | This work |
| DS21 | trpC2 aprE ::kan(lacI $P_{spac}$-repN/oriN) dnaA $^{R202A}$ ::cat | HM1108 | pDS45 | This work |
| DS22 | trpC2 aprE ::kan(lacI $P_{spac}$-repN/oriN) dnaA $^{R206A}$ ::cat | HM1108 | pDS4 | This work |
| DS23 | trpC2 aprE ::kan(lacI $P_{spac}$-repN/oriN) dnaA $^{N187A}$ ::cat | HM1108 | pDS47 | This work |
| DS25 | trpC2 aprE ::kan(lacI $P_{spac}$-repN/oriN) dnaA $^{F128A}$ ::cat | HM1108 | pDS55 | This work |
| DS26 | trpC2 aprE ::kan(lacI $P_{spac}$-repN/oriN) dnaA $^{F218A}$ ::cat | HM1108 | pDS56 | This work |
| DS27 | trpC2 aprE ::kan(lacI $P_{spac}$-repN/oriN) dnaA $^{R321A}$ ::cat | HM1108 | pDS57 | This work |
| DS34 | trpC2 aprE ::kan(lacI $P_{spac}$-repN/oriN) dnaA $^{L269A}$ ::cat | HM1108 | pDS66 | This work |
| DS50 | trpC2 aprE ::kan(lacI $P_{spac}$-repN/oriN) dnaA $^{H231A}$ ::cat | HM1108 | pCB3 | This work |
| DS51 | trpC2 aprE ::kan(lacI $P_{spac}$-repN/oriN) dnaA $^{G317Q}$ ::cat | HM1108 | pCB6 | This work |
| DS52 | trpC2 aprE ::kan(lacI $P_{spac}$-repN/oriN) dnaA $^{T225A}$ ::cat | HM1108 | pDS118 | This work |
| DS53 | trpC2 aprE ::kan(lacI $P_{spac}$-repN/oriN) dnaA $^{K222A}$ ::cat | HM1108 | pDS116 | This work |
| DS54 | trpC2 aprE ::kan(lacI $P_{spac}$-repN/oriN) dnaA $^{Q224A}$ ::cat | HM1108 | pDS117 | This work |
| DS56 | trpC2 aprE ::kan(lacI $P_{spac}$-repN/oriN) dnaA $^{R264A}$ ::cat | HM1108 | pDS123 | This work |
| DS57 | trpC2 spoIIIJ (359°)::(oriN kan tet ) ΔdnaA ::zeo amyE ::spc (xylR $P_{xyl}$-dnaAchi$^{I190A}$ ) | HM1423 | TR244 | This work |
| DS58 | trpC2 spoIIIJ (359°)::(oriN kan tet ) ΔdnaA ::zeo amyE ::spc (xylR $P_{xyl}$-dnaAchi$^{K222A}$ ) | HM1423 | TR262 | This work |
| DS60 | trpC2 spoIIIJ (359°)::(oriN kan tet ) ΔdnaA ::zeo amyE ::spc (xylR $P_{xyl}$-dnaAchi$^{R264A}$ ) | HM1423 | TR313 | This work |
| DS61 | trpC2 spoIIIJ (359°)::(oriN kan tet ) ΔdnaA ::zeo amyE ::spc (xylR $P_{xyl}$-dnaAchi$^{R202A}$ ) | HM1423 | TR480 | This work |
| DS62 | trpC2 spoIIIJ (359°)::(oriN kan tet ) ΔdnaA ::zeo amyE ::spc (xylR $P_{xyl}$-dnaAchi$^{R206A}$ ) | HM1423 | TR481 | This work |
| DS64 | trpC2 spoIIIJ (359°)::(oriN kan tet ) ΔdnaA ::zeo amyE ::spc (xylR $P_{xyl}$-dnaAchi$^{L269A}$ ) | HM1423 | TR483 | This work |
| DS65 | trpC2 spoIIIJ (359°)::(oriN kan tet ) ΔdnaA ::zeo amyE ::spc (xylR $P_{xyl}$-dnaAchi$^{F218A}$ ) | HM1423 | TR486 | This work |
| DS66 | trpC2 spoIIIJ (359°)::(oriN kan tet ) ΔdnaA ::zeo amyE ::spc (xylR $P_{xyl}$-dnaAchi$^{R321A}$ ) | HM1423 | TR488 | This work |
| DS68 | trpC2 spoIIIJ (359°)::(oriN kan tet ) ΔdnaA ::zeo amyE ::spc (xylR $P_{xyl}$-dnaAchi ) | HM1423 | HM1683 | This work |
| HM1108 | trpC2 aprE ::kan (lacI $P_{spac}$-repN/oriN ) | | | Richardson *et al.* 2016 |
| HM1358 | trpC2 aprE ::kan ($P_{spac}$-$P_{xyl}$-xylR ) | HM715 | pHM510 | This work |
| HM1423 | trpC2 spoIIIJ( 359°) ::(oriN kan tet) ΔdnaA ::zeo | | | Murray and Koh 2014 |
| HM1552 | trpC2 aprE ::kan (lacI $P_{spac}$-repN/oriN ) incC ::cat | | | Richardson *et al.* 2016 |
| HM1554 | trpC2 aprE ::kan (lacI $P_{spac}$-repN/oriN ) incC (ΔDnaA-box#6/7)::cat | HM1108 | pTR95 | This work |
| HM1560 | trpC2 aprE ::kan (lacI $P_{spac}$-repN/oriN ) incC (ΔDnaA-box#1/2/3/4/7)::cat | HM1108 | pTR199 | This work |
| HM1571 | trpC2 aprE ::kan (lacI $P_{spac}$-repN/oriN ) incC (ΔDnaA-box#[6/7]$^{rev}$)::cat | HM1108 | pTR127 | This work |
| HM1574 | trpC2 aprE ::kan (lacI $P_{spac}$-repN/oriN ) incC (ΔDnaA-box#6$^{rev}$)::cat | HM1108 | pTR189 | This work |
| HM1607 | trpC2 aprE ::kan (lacI $P_{spac}$-repN/oriN ) incC (ΔDnaA-box#1/2/3/4/5/7)::cat | HM1108 | pTR158 | This work |
| HM1643 | trpC2 aprE ::kan (lacI $P_{spac}$-repN/oriN ) incC (ΔDnaA-box#1/4/5/7)::cat | HM1108 | pTR324 | This work |
| HM1651 | trpC2 aprE ::kan (lacI $P_{spac}$-repN/oriN ) incC (ΔDnaA-box#7)::cat | HM1108 | pTR90 | This work |
| HM1671 | trpC2 aprE ::kan (lacI $P_{spac}$-repN/oriN ) incC (ΔDnaA-box#2/3/4/5)::cat | HM1108 | pTR393 | This work |
| HM1683 | trpC2 aprE ::kan (lacI $P_{spac}$-repN/oriN ) amyE ::spc (xylR $P_{xyl}$-dnaAchi ) | HM1108 | pHM610 | This work |
| HM1694 | trpC2 aprE ::kan (lacI $P_{spac}$-repN/oriN ) incC$^{art}$ (DnaA-box#6/7)::cat amyE ::spc (xylR $P_{xyl}$-dnaAchi ) | HM1683 | pTR211 | This work |
| HM1834 | trpC2 aprE ::kan (lacI $P_{spac}$-repN/oriN ) incC$^{art}$ (DnaA-box#Tm$^{45}$/6/7)::cat | HM1108 | pTR664 | This work |
| HM1844 | trpC2 incC$^{art}$ ::cat (DnaA-box#6/7 amyE ::spc ($P_{xyl}$-dnaA-dnaN ) | TR723 | TR684 | This work |
| HM1845 | trpC2 incC ::cat amyE ::spc ($P_{xyl}$-dnaA-dnaN ) | TR723 | TR684 | This work |
| HM1846 | trpC2 sacA ::cat ($P_{hbs}$-hbs-gfp ) | HM715 | BWX2006 (Wang *et al.* 2014) | This work |
| HM1854 | trpC2 incC ::cat amyE ::spc ($P_{xyl}$-dnaA-dnaN ) aprE ::kan ($P_{spac}$-$P_{xylR}$-xylR ) | HM1845 | HM1358 | This work |
| HM1856 | trpC2 incC$^{art}$ ::cat (DnaA-box#6/7 amyE ::spc ($P_{xyl}$-dnaA-dnaN ) aprE ::kan ($P_{spac}$-$P_{xylR}$-xylR ) | HM1844 | HM1358 | This work |
| HM1858 | trpC2 incC ::cat amyE ::spc ($P_{xyl}$-dnaA-dnaN ) aprE ::kan ($P_{spac}$-$P_{xylR}$-xylR ) Δsda ::tet | HM1854 | JWV44 (Veening *et al.* 2009) | This work |
| HM1860 | trpC2 incC$^{art}$ ::cat (DnaA-box#6/7 amyE ::spc ($P_{xyl}$-dnaA-dnaN ) aprE ::kan ($P_{spac}$-$P_{xylR}$-xylR ) Δsda ::tet | HM1856 | JWV44 (Veening *et al.* 2009) | This work |
| HM1862 | trpC2 sacA ::cat'-tet-'cat ($P_{hbs}$-hbs-gfp ) | HM1846 | pCm::Tc (Steinmetz & Richter 1994) | This work |
| HM1863 | trpC2 incC ::cat amyE ::spc ($P_{xyl}$-dnaA-dnaN ) aprE ::kan ($P_{spac}$-$P_{xylR}$-xylR ) sacA ::cat'-tet-'cat ($P_{hbs}$-hbs-gfp ) | HM1854 | HM1862 | This work |
| HM1864 | trpC2 incC$^{art}$ ::cat (DnaA-box#6/7 amyE ::spc ($P_{xyl}$-dnaA-dnaN ) aprE ::kan ($P_{spac}$-$P_{xylR}$-xylR ) sacA ::cat'-tet-'cat ($P_{hbs}$-hbs-gfp ) | HM1856 | HM1862 | This work |
| OH21 | trpC2 aprE ::kan (lacI $P_{spac}$-repN/oriN ) incC (ΔDnaA-box#1)::cat | HM1108 | pTR85 | This work |
| OH61 | trpC2 aprE ::kan (lacI $P_{spac}$-repN/oriN ) incC (ΔDnaA-box#1/2)::cat | HM1108 | pTR130 | This work |
| OH62 | trpC2 aprE ::kan (lacI $P_{spac}$-repN/oriN ) incC (ΔDnaA-box#1/2/3)::cat | HM1108 | pTR131 | This work |
| OH63 | trpC2 aprE ::kan (lacI $P_{spac}$-repN/oriN ) incC (ΔDnaA-box#1/2/3/4)::cat | HM1108 | pTR132 | This work |
| OH64 | trpC2 aprE ::kan (lacI $P_{spac}$-repN/oriN ) incC (ΔDnaA-box#1/2/3/4/5)::cat | HM1108 | pTR133 | This work |
| OH70 | trpC2 aprE ::kan (lacI $P_{spac}$-repN/oriN ) incC$^{art}$ (DnaA-box#CR$^{44}$/6/7)::cat | HM1108 | pOH040 | This work |
| TR122 | trpC2 aprE ::kan (lacI $P_{spac}$-repN/oriN ) incC (ΔDnaA-box#1/3/4/5)::cat | HM1108 | pTR361 | This work |
| TR123 | trpC2 aprE ::kan (lacI $P_{spac}$-repN/oriN ) incC (ΔDnaA-box#1/2/4/5)::cat | HM1108 | pTR362 | This work |
| TR124 | trpC2 aprE ::kan (lacI $P_{spac}$-repN/oriN ) incC (ΔDnaA-box#1/4/5)::cat | HM1108 | pTR363 | This work |
| TR184 | trpC2 aprE ::kan (lacI $P_{spac}$-repN/oriN ) incC$^{art}$ (DnaA-box#CR$^{75}$/6/7)::cat | HM1108 | pTR560 | This work |

# Appendix Table 1. Strains

| | | | | |
|---|---|---|---|---|
| TR186 | trpC2 aprE::kan (lacI $P_{spac}$-repN/oriN) $incC^{art}$ (DnaA-box#$CR^{55}$/6/7)::cat | HM1108 | pTR562 | This work |
| TR203 | trpC2 aprE::kan (lacI $P_{spac}$-repN/oriN) $incC^{x\_art}$ (DnaA-box#$CR^{132}$/6/7)::cat | HM1108 | pTR641 | This work |
| TR206 | trpC2 aprE::kan (lacI $P_{spac}$-repN/oriN) $incC^{x\_art}$ (DnaA-box#$CR^{297}$/6/7)::cat | HM1108 | pTR644 | This work |
| TR208 | trpC2 aprE::kan (lacI $P_{spac}$-repN/oriN) $incC^{x\_art}$ (DnaA-box#$CR^{462}$/6/7)::cat | HM1108 | pTR646 | This work |
| TR209 | trpC2 aprE::kan (lacI $P_{spac}$-repN/oriN) $incC^{x\_art}$ (DnaA-box#6/7)::cat | HM1108 | pTR647 | This work |
| TR210 | trpC2 aprE::kan (lacI $P_{spac}$-repN/oriN) $incC^{x\_art}$ (DnaA-box#$CR^{44}$/6/7)::cat | HM1108 | pTR648 | This work |
| TR215 | trpC2 aprE::kan (lacI $P_{spac}$-repN/oriN) $incC^{art}$ ::cat | HM1108 | pTR653 | This work |
| TR241 | trpC2 aprE::kan (lacI $P_{spac}$-repN/oriN) $incC^{art}$ (DnaA-box#$Tm^{45}$/6/7)::cat amyE::spc (xylR $P_{xyl}$-dnaAchi) | HM1683 | pTR664 | This work |
| TR244 | trpC2 aprE::kan (lacI $P_{spac}$-repN/oriN) $incC^{art}$ (DnaA-box#$Tm^{45}$/6/7)::cat amyE::spc (xylR $P_{xyl}$-$dnaAchi^{I190A}$) | HM1834 | pTR504 | This work |
| TR262 | trpC2 aprE::kan (lacI $P_{spac}$-repN/oriN) $incC^{art}$ (DnaA-box#$Tm^{45}$/6/7)::cat amyE::spc (xylR $P_{xyl}$-$dnaAchi^{K222A}$) | HM1834 | pTR621 | This work |
| TR265 | trpC2 aprE::kan (lacI $P_{spac}$-repN/oriN) $incC^{art}$ (DnaA-box#$Tm^{45}$/6/7)::cat amyE::spc (xylR $P_{xyl}$-$dnaAchi^{T225A}$) | HM1834 | pTR622 | This work |
| TR313 | trpC2 aprE::kan (lacI $P_{spac}$-repN/oriN) $incC^{art}$ (DnaA-box#$Tm^{45}$/6/7)::cat amyE::spc (xylR $P_{xyl}$-$dnaAchi^{R264A}$) | HM1834 | pTR699 | This work |
| TR324 | trpC2 aprE::kan (lacI $P_{spac}$-repN/oriN) $incC^{art}$ (DnaA-box#$CR^{49}$/6/7)::cat | HM1108 | pTR732 | This work |
| TR325 | trpC2 aprE::kan (lacI $P_{spac}$-repN/oriN) $incC^{art}$ (DnaA-box#$CR^{39}$/6/7)::cat | HM1108 | pTR733 | This work |
| TR326 | trpC2 aprE::kan (lacI $P_{spac}$-repN/oriN) $incC^{art}$ (DnaA-box#$CR^{33}$/6/7)::cat | HM1108 | pTR734 | This work |
| TR328 | trpC2 aprE::kan (lacI $P_{spac}$-repN/oriN) $incC^{art}$ (DnaA-box#$CR^{60}$/6/7)::cat | HM1108 | pTR748 | This work |
| TR329 | trpC2 aprE::kan (lacI $P_{spac}$-repN/oriN) $incC^{art}$ (DnaA-box#$CR^{65}$/6/7)::cat | HM1108 | pTR749 | This work |
| TR330 | trpC2 aprE::kan (lacI $P_{spac}$-repN/oriN) $incC^{art}$ (DnaA-box#$CR^{70}$/6/7)::cat | HM1108 | pTR750 | This work |
| TR411 | trpC2 aprE::kan (lacI $P_{spac}$-repN/oriN) incC (ΔDnaA-box#3/4/5)::cat | HM1108 | pTR836 | This work |
| TR412 | trpC2 aprE::kan (lacI $P_{spac}$-repN/oriN) incC (ΔDnaA-box#2/4/5)::cat | HM1108 | pTR837 | This work |
| TR452 | trpC2 aprE::kan (lacI $P_{spac}$-repN/oriN) incC (ΔDnaA-box#4/5)::cat | HM1108 | pTR865 | This work |
| TR480 | trpC2 aprE::kan (lacI $P_{spac}$-repN/oriN) $incC^{art}$ (DnaA-box#$Tm^{45}$/6/7)::cat amyE::spc (xylR $P_{xyl}$-$dnaAchi^{R202A}$) | HM1834 | pTR881 | This work |
| TR481 | trpC2 aprE::kan (lacI $P_{spac}$-repN/oriN) $incC^{art}$ (DnaA-box#$Tm^{45}$/6/7)::cat amyE::spc (xylR $P_{xyl}$-$dnaAchi^{R206A}$) | HM1834 | pTR882 | This work |
| TR483 | trpC2 aprE::kan (lacI $P_{spac}$-repN/oriN) $incC^{art}$ (DnaA-box#$Tm^{45}$/6/7)::cat amyE::spc (xylR $P_{xyl}$-$dnaAchi^{L269A}$) | HM1834 | pTR885 | This work |
| TR486 | trpC2 aprE::kan (lacI $P_{spac}$-repN/oriN) $incC^{art}$ (DnaA-box#$Tm^{45}$/6/7)::cat amyE::spc (xylR $P_{xyl}$-$dnaAchi^{F218A}$) | HM1834 | pTR883 | This work |
| TR488 | trpC2 aprE::kan (lacI $P_{spac}$-repN/oriN) $incC^{art}$ (DnaA-box#$Tm^{45}$/6/7)::cat amyE::spc (xylR $P_{xyl}$-$dnaAchi^{R321A}$) | HM1834 | pTR887 | This work |
| TR657 | trpC2 aprE::kan (lacI $P_{spac}$-repN/oriN) $incC^{art}$ (DnaA-box#$CL^{86}$/6/7)::cat | HM1108 | pTR991 | This work |
| TR671 | trpC2 aprE::kan (lacI $P_{spac}$-repN/oriN) amyE::spc ($P_{xyl}$-dnaA) | HM1108 | see Methods | This work |
| TR672 | trpC2 aprE::kan (lacI $P_{spac}$-repN/oriN) amyE::spc (xylR $P_{xyl}$-dnaA-dnaN) | HM1108 | see Methods | This work |
| TR674 | trpC2 aprE::kan (lacI $P_{spac}$-repN/oriN) amyE::spc ($P_{xyl}$-dnaA) $incC^{art}$ (DnaA-box#6/7)::cat | TR671 | pTR211 | This work |
| TR684 | trpC2 aprE::kan (lacI $P_{spac}$-repN/oriN) amyE::spc ($P_{xyl}$-dnaA-dnaN) $incC^{art}$ (DnaA-box#6/7)::cat | TR672 | pTR211 | This work |
| TR690 | trpC2 aprE::kan (lacI $P_{spac}$-repN/oriN) $incC^{art}$ (DnaA-box#$CL^{91}$/6/7)::cat | HM1108 | pTR1012 | This work |
| TR691 | trpC2 aprE::kan (lacI $P_{spac}$-repN/oriN) $incC^{art}$ (DnaA-box#$CL^{96}$/6/7)::cat | HM1108 | pTR1013 | This work |
| TR698 | trpC2 aprE::kan (lacI $P_{spac}$-repN/oriN) amyE::spc ($P_{xyl}$-dnaA-dnaN) $incC^{art}$ (DnaA-box#$CR^{44}$6/7)::cat | TR672 | pOH40 | This work |
| TR707 | trpC2 aprE::kan (lacI $P_{spac}$-repN/oriN) $incC^{art}$ (DnaA-box#$CR^{91}$/6/7)::cat | HM1108 | pTR1055 | This work |
| TR708 | trpC2 aprE::kan (lacI $P_{spac}$-repN/oriN) $incC^{art}$ (DnaA-box#$CR^{86}$/6/7)::cat | HM1108 | pTR1054 | This work |
| TR709 | trpC2 aprE::kan (lacI $P_{spac}$-repN/oriN) $incC^{art}$ (DnaA-box#$CR^{80}$/6/7)::cat | HM1108 | pTR1053 | This work |
| TR710 | trpC2 aprE::kan (lacI $P_{spac}$-repN/oriN) $incC^{art}$ (DnaA-box#$CR^{28}$/6/7)::cat | HM1108 | pTR1051 | This work |
| TR711 | trpC2 aprE::kan (lacI $P_{spac}$-repN/oriN) $incC^{art}$ (DnaA-box#$CR^{22}$/6/7)::cat | HM1108 | pTR1052 | This work |
| TR723 | trpC2 amyE::spc ($P_{xyl}$-dnaA-dnaN) | HM715 | TR672 | This work |

# Appendix Table 2. Plasmids

| Plasmid | Genotype | Quickchange plasmid | Quickchange oligos | Subcloning backbone | Subcloning insert | Subcloning enzymes | Reference |
|---|---|---|---|---|---|---|---|
| pCB2 | *bla 'dnaA* $^{H231A}$ *incC dnaN cat recF* | pHM327 | oCB5/oCB6 | | | | This work |
| pCB3 | *bla 'dnaA* $^{H231A}$ *incC dnaN cat recF* | | | pHM327 | pCB2 | PacI/PflMI | This work |
| pCB5 | *bla 'dnaA* $^{G317Q}$ *incC dnaN cat recF* | pHM327 | oCB11/oCB12 | | | | This work |
| pCB6 | *bla 'dnaA* $^{G317Q}$ *incC dnaN cat recF* | | | pHM327 | pCB5 | PacI/PflMI | This work |
| pDS3 | *bla 'dnaA* $^{R206A}$ *incC dnaN cat recF* | pHM327 | oDS3/oDS4 | | | | This work |
| pDS4 | *bla 'dnaA* $^{R206A}$ *incC dnaN cat recF* | | | pHM327 | pDS3 | PacI/PflMI | This work |
| pDS12 | *bla 'dnaA* $^{E183A}$ *incC dnaN cat recF* | pHM327 | oDS7/oDS8 | | | | This work |
| pDS22 | *bla 'dnaA* $^{E183A}$ *incC dnaN cat recF* | | | pHM327 | pDS12 | PacI/PflMI | This work |
| pDS45 | *bla 'dnaA* $^{R202A}$ *incC dnaN cat recF* | | | pHM327 | pTS15 | PacI/PflMI | This work |
| pDS47 | *bla 'dnaA* $^{N187A}$ *incC dnaN cat recF* | | | pHM327 | pTS10 | PacI/PflMI | This work |
| pDS50 | *bla 'dnaA* $^{I190A}$ *incC dnaN cat recF* | | | pHM327 | pTS2 | PacI/PflMI | This work |
| pDS52 | *bla 'dnaA* $^{F128A}$ *incC dnaN cat recF* | pHM327 | oDS45/oDS46 | | | | This work |
| pDS53 | *bla 'dnaA* $^{F218A}$ *incC dnaN cat recF* | pHM327 | oDS47/oDS48 | | | | This work |
| pDS54 | *bla 'dnaA* $^{R321A}$ *incC dnaN cat recF* | pHM327 | oDS51/oDS52 | | | | This work |
| pDS55 | *bla 'dnaA* $^{F128A}$ *incC dnaN cat recF* | | | pHM327 | pDS52 | PacI/PflMI | This work |
| pDS56 | *bla 'dnaA* $^{F218A}$ *incC dnaN cat recF* | | | pHM327 | pDS53 | PacI/PflMI | This work |
| pDS57 | *bla 'dnaA* $^{R321A}$ *incC dnaN cat recF* | | | pHM327 | pDS54 | PacI/PflMI | This work |
| pDS65 | *bla 'dnaA* $^{L269A}$ *incC dnaN cat recF* | pHM327 | oDS49/oDS50 | | | | This work |
| pDS66 | *bla 'dnaA* $^{L269A}$ *incC dnaN cat recF* | | | pHM327 | pDS65 | PacI/PflMI | This work |
| pDS116 | *bla 'dnaA* $^{K222A}$ *incC dnaN cat recF* | | | pHM327 | pTS4 | PacI/PflMI | This work |
| pDS117 | *bla 'dnaA* $^{Q224A}$ *incC dnaN cat recF* | | | pHM327 | pTS5 | PacI/PflMI | This work |
| pDS118 | *bla 'dnaA* $^{T225A}$ *incC dnaN cat recF* | | | pHM327 | pTS6 | PacI/PflMI | This work |
| pDS123 | *bla 'dnaA* $^{R264A}$ *incC dnaN cat recF* | | | pHM327 | pTS7 | PacI/PflMI | This work |
| pHM293 | *bla phleo* | see Methods | | | | | This work |
| pHM294 | *bla phleo* $P_{xylR}$-*xylR* | see Methods | | | | | This work |
| pHM327 | *bla 'dnaA incC dnaN cat recF* | | | | | | Scholefield, 2012 |
| pHM367 | *bla 'dnaA incC dnaN'* | see Methods | | | | | This work |
| pHM510 | *bla aprE' kan lacI* $P_{spac}$-$P_{xylR}$-*xylR 'aprE* | see Methods | | | | | This work |
| pHM610 | *bla amyE'* $P_{xyl}$-*dnaAchi xylR spc 'amyE* | see Methods | | | | | This work |
| pKM322 | *bla amyE'* $P_{xyl}$-*dnaA-gfp xylR spc 'amyE* | | | | | | Wagner, 2009 |
| pOH18 | *bla 'dnaA incC* $^{x\_art}$ (DnaA-box#Tm$^{45}$/6/7) *dnaN'* | pTR424 | oOH28/oOH29 | | | | This work |
| pOH40 | *bla 'dnaA incC* $^{art}$ (DnaA-box#CR$^{44}$/6/7) *dnaN cat recF* | | | pHM327 | pTR552 | BglII/FspAI | This work |
| pTR27 | *bla 'dnaA incC* (ΔDnaA-box#1) *dnaN'* | pHM367 | oTR118/oTR119 | | | | This work |
| pTR29 | *bla 'dnaA incC* (ΔDnaA-box#3) *dnaN'* | pHM367 | oTR82/oTR83 | | | | This work |
| pTR33 | *bla 'dnaA incC* (ΔDnaA-box#7) *dnaN'* | pHM367 | oTR90/oTR91 | | | | This work |
| pTR35 | *bla lacZ'* DnaA-box#6 AT$^{27mer}$ | pTR42 | oTR102/oTR103 | | | | This work |
| pTR42 | *bla lacZ'* DnaA-box#6 | pUC18 | oTR96/oTR97 | | | | This work |
| pTR60 | *bla 'dnaA incC* (ΔDnaA-box#5/7) *dnaN'* | pTR33 | oTR134/oTR135 | | | | This work |
| pTR61 | *bla 'dnaA incC* (ΔDnaA-box#6/7) *dnaN'* | pHM367 | oTR136/oTR137 | | | | This work |
| pTR85 | *bla 'dnaA incC* (ΔDnaA-box#1) *dnaN cat recF* | | | pHM327 | pTR27 | BglII/FspAI | This work |
| pTR90 | *bla 'dnaA incC* (ΔDnaA-box#7) *dnaN cat recF* | | | pHM327 | pTR33 | BglII/FspAI | This work |
| pTR95 | *bla 'dnaA incC* (ΔDnaA-box#6/7) *dnaN cat recF* | | | pHM327 | pTR61 | BglII/FspAI | This work |
| pTR114 | *bla lacZ'* DnaA-box#6 DnaA-trios AT$^{27mer}$ | pTR35 | oTR334/oTR335 | | | | This work |
| pTR116 | *bla 'dnaA incC* (DnaA-box#6$^{rev}$) *dnaN'* | pHM367 | oTR217/oTR218 | | | | This work |
| pTR117 | *bla 'dnaA incC* (DnaA-box#[6/7]$^{rev}$) *dnaN'* | pTR116 | oTR219/oTR220 | | | | This work |
| pTR120 | *bla 'dnaA incC* (ΔDnaA-box#1/2) *dnaN'* | pTR27 | oTR227/oTR228 | | | | This work |
| pTR121 | *bla 'dnaA incC* (ΔDnaA-box#1/2/3) *dnaN'* | pTR120 | oTR229/oTR230 | | | | This work |
| pTR122 | *bla 'dnaA incC* (ΔDnaA-box#1/2/3/4) *dnaN'* | pTR121 | oTR231/oTR232 | | | | This work |
| pTR123 | *bla 'dnaA incC* (ΔDnaA-box#1/2/3/4/5) *dnaN'* | pTR122 | oTR233/oTR234 | | | | This work |
| pTR127 | *bla 'dnaA incC* (ΔDnaA-box#[6/7]$^{rev}$) *dnaN cat recF* | | | pHM327 | pTR117 | BglII/FspAI | This work |
| pTR130 | *bla 'dnaA incC* (ΔDnaA-box#1/2) *dnaN cat recF* | | | pHM327 | pTR120 | BglII/FspAI | This work |
| pTR131 | *bla 'dnaA incC* (ΔDnaA-box#1/2/3) *dnaN cat recF* | | | pHM327 | pTR121 | BglII/FspAI | This work |
| pTR132 | *bla 'dnaA incC* (ΔDnaA-box#1/2/3/4) *dnaN cat recF* | | | pHM327 | pTR122 | BglII/FspAI | This work |
| pTR133 | *bla 'dnaA incC* (ΔDnaA-box#1/2/3/4/5) *dnaN cat recF* | | | pHM327 | pTR123 | BglII/FspAI | This work |
| pTR155 | *bla 'dnaA incC* (ΔDnaA-box#1/2/3/4/5/7) *dnaN'* | pTR123 | oTR90/oTR153 | | | | This work |
| pTR158 | *bla 'dnaA incC* (ΔDnaA-box#1/2/3/4/5/7) *dnaN cat recF* | | | pHM327 | pTR155 | BglII/FspAI | This work |

# Appendix Table 2. Plasmids

| | | | | | | | |
|---|---|---|---|---|---|---|---|
| pTR189 | *bla 'dnaA incC* (DnaA-box#6$^{rev}$) *dnaN cat recF* | | | pHM327 | pTR116 | BglII/FspAI | This work |
| pTR198 | *bla 'dnaA incC* (ΔDnaA-box#1/2/3/4/7) *dnaN'* | pTR155 | oTR366/oTR367 | | | | This work |
| pTR199 | *bla 'dnaA incC* (ΔDnaA-box#1/2/3/4/7) *dnaN cat recF* | | | pHM327 | pTR198 | BglII/FspAI | This work |
| pTR204 | *bla lacZ'* DnaA-box#6/7 DnaA-trios AT$^{27mer}$ | pTR114 | oTR374/oTR375 | | | | This work |
| pTR207 | *bla 'dnaA incC*$^{art}$ (DnaA-box#6/7) *dnaN'* | see Methods | | | | | This work |
| pTR211 | *bla 'dnaA incC*$^{art}$ (DnaA-box#6/7) *dnaN cat recF* | | | pHM327 | pTR207 | BglII/FspAI | This work |
| pTR213 | *bla 'dnaA incC dnaN'* ΔDnaA-box(nt866-874) | pHM367 | oTR393/oTR394 | | | | This work |
| pTR214 | *bla 'dnaA incC dnaN'* ΔDnaA-box(nt866-874) ΔDnaA-box(nt772-780) | pTR213 | oTR404/oTR405 | | | | This work |
| pTR228 | *bla 'dnaA incC*$^{art}$ (DnaA-box#/6/7) *dnaN'* ΔDnaA-box(nt866-874) ΔDnaA-box(nt772-780) | | | pTR214 | pTR207 | BglII/FspAI | This work |
| pTR307 | *bla 'dnaA incC* (ΔDnaA-box#1/5/7) *dnaN'* | pTR60 | oTR118/oTR119 | | | | This work |
| pTR322 | *bla 'dnaA incC* (ΔDnaA-box#1/4/5/7) *dnaN'* | pTR307 | oTR483/oTR484 | | | | This work |
| pTR324 | *bla 'dnaA incC* (ΔDnaA-box#1/4/5/7) *dnaN cat recF* | | | pHM327 | pTR322 | BglII/FspAI | This work |
| pTR327 | *bla 'dnaA incC* (ΔDnaA-box#2/3) *dnaN'* | pTR29 | oTR488/oTR489 | | | | This work |
| pTR354 | *bla 'dnaA incC* (ΔDnaA-box#1/3/4/5) *dnaN'* | pTR123 | oTR519/oTR520 | | | | This work |
| pTR355 | *bla 'dnaA incC* (ΔDnaA-box#1/2/4/5) *dnaN'* | pTR123 | oTR521/oTR522 | | | | This work |
| pTR356 | *bla 'dnaA incC* (ΔDnaA-box#1/4/5) *dnaN'* | pTR322 | oTR523/oTR524 | | | | This work |
| pTR361 | *bla 'dnaA incC* (ΔDnaA-box#1/3/4/5) *dnaN cat recF* | | | pHM327 | pTR354 | BglII/FspAI | This work |
| pTR362 | *bla 'dnaA incC* (ΔDnaA-box#1/2/4/5) *dnaN cat recF* | | | pHM327 | pTR355 | BglII/FspAI | This work |
| pTR363 | *bla 'dnaA incC* (ΔDnaA-box#1/4/5) *dnaN cat recF* | | | pHM327 | pTR356 | BglII/FspAI | This work |
| pTR365 | *bla 'dnaA incC* (ΔDnaA-box#2/3/4) *dnaN'* | pTR327 | oTR231/oTR232 | | | | This work |
| pTR392 | *bla 'dnaA incC* (ΔDnaA-box#2/3/4/5) *dnaN'* | pTR365 | oTR233/oTR234 | | | | This work |
| pTR393 | *bla 'dnaA incC* (ΔDnaA-box#2/3/4/5) *dnaN cat recF* | | | pHM327 | pTR392 | BglII/FspAI | This work |
| pTR424 | *bla 'dnaA incC*$^{art}$ (DnaA-box#/6/7) *dnaN'* ΔDnaA-box(nt866-874) ΔDnaA-box(nt772-780) Δnt1654-1754 | pTR228 | oTR604/oTR605 | | | | This work |
| pTR504 | *bla amyE'* $P_{xyl}$-*dnaAchi*$^{I190A}$ *xylR spc 'amyE* | pHM610 | oTR415/oTR416 | | | | This work |
| pTR548 | *bla 'dnaA incC*$^{art}$ (DnaA-box#CR$^{75}$/6/7) *dnaN'* | pTR424 | oTR1062/oTR1063 | | | | This work |
| pTR550 | *bla 'dnaA incC*$^{art}$ (DnaA-box#CR$^{55}$/6/7) *dnaN'* | pTR424 | oTR1066/oTR1067 | | | | This work |
| pTR552 | *bla 'dnaA incC*$^{art}$ (DnaA-box#CR$^{44}$/6/7) *dnaN'* | pTR424 | oOH51/oOH52 | | | | This work |
| pTR560 | *bla 'dnaA incC*$^{art}$ (DnaA-box#CR$^{75}$/6/7) *dnaN cat recF* | | | pHM327 | pTR548 | BglII/FspAI | This work |
| pTR562 | *bla 'dnaA incC*$^{art}$ (DnaA-box#CR$^{55}$/6/7) *dnaN cat recF* | | | pHM327 | pTR550 | BglII/FspAI | This work |
| pTR587 | *bla 'dnaA incC*$^{art}$ (DnaA-box#CR$^{49}$/6/7) *dnaN'* | pTR424 | oTR1177/oTR1178 | | | | This work |
| pTR588 | *bla 'dnaA incC*$^{art}$ (DnaA-box#CR$^{39}$/6/7) *dnaN'* | pTR424 | oTR1179/oTR1180 | | | | This work |
| pTR589 | *bla 'dnaA incC*$^{art}$ (DnaA-box#CR$^{33}$/6/7) *dnaN'* | pTR424 | oTR1181/oTR1182 | | | | This work |
| pTR595 | *bla 'dnaA incC*$^{x-art}$ (DnaA-box#CR$^{132}$/6/7) *dnaN'* | pTR607 | oTR1189/oTR1190 | | | | This work |
| pTR598 | *bla 'dnaA incC*$^{x-art}$ (DnaA-box#CR$^{297}$/6/7) *dnaN'* | pTR607 | oTR1195/oTR1196 | | | | This work |
| pTR600 | *bla 'dnaA incC*$^{x-art}$ (DnaA-box#CR$^{462}$/6/7) *dnaN'* | pTR607 | oTR1199/oTR1200 | | | | This work |
| pTR606 | *bla 'dnaA incC*$^{art}$ (DnaA-box#CR$^{44}$/6/7) *dnaN'* Δnt3431-250 | pTR552 (see Methods) | oTR1201/oTR1202 | | | | This work |
| pTR607 | *bla 'dnaA incC*$^{x-art}$ (DnaA-box#6/7) *dnaN'* | pTR608 | oTR1221/oTR1222 | | | | This work |
| pTR608 | *bla 'dnaA incC*$^{x-art}$ (DnaA-box#CR$^{44}$6/7) *dnaN'* | see Methods | | | | | This work |
| pTR610 | *bla 'dnaA incC*$^{art}$ (DnaA-box#/7) *dnaN'* | pTR615 | oTR1209/oTR1210 | | | | This work |
| pTR611 | *bla 'dnaA incC*$^{art}$ *dnaN'* | pTR610 | oTR1211/oTR1212 | | | | This work |
| pTR612 | *bla 'dnaA incC*$^{art}$ (ΔGC-rich) *dnaN'* | pTR611 | oTR1213/oTR1214 | | | | This work |
| pTR613 | *bla 'dnaA incC*$^{art}$ (ΔGC-rich ΔDnaA-trios) *dnaN'* | pTR612 | oTR1215/oTR1216 | | | | This work |
| pTR614 | *bla 'dnaA incC*$^{art}$ (ΔGC-rich ΔDnaA-trios ΔAT-rich) *dnaN'* | pTR613 | oTR1217/oTR1218 | | | | This work |
| pTR615 | *bla 'dnaA incC*$^{art}$ (DnaA-box#/6/7) *dnaN'* Δnt1615-1738 | pTR424 | oTR1219/oTR1220 | | | | This work |
| pTR621 | *bla amyE'* $P_{xyl}$-*dnaAchi*$^{K222A}$ *xylR spc 'amyE* | pHM610 | oTR368/oTR369 | | | | This work |
| pTR622 | *bla amyE'* $P_{xyl}$-*dnaAchi*$^{T225A}$ *xylR spc 'amyE* | pHM610 | oTR372/oTR373 | | | | This work |
| pTR634 | *bla 'dnaA incC dnaN cat recF* Δnt5617-6160 | pHM327 (see Methods) | oTR1251/oTR1252 | | | | |
| pTR641 | *bla 'dnaA incC*$^{x-art}$ (DnaA-box#CR$^{132}$/6/7) *dnaN cat recF* | | | pTR634 | pTR595 | BglII/FspAI | This work |
| pTR644 | *bla 'dnaA incC*$^{x-art}$ (DnaA-box#CR$^{297}$/6/7) *dnaN cat recF* | | | pTR634 | pTR598 | BglII/FspAI | This work |
| pTR646 | *bla 'dnaA incC*$^{x-art}$ (DnaA-box#CR$^{462}$/6/7) *dnaN cat recF* | | | pTR634 | pTR600 | BglII/FspAI | This work |
| pTR647 | *bla 'dnaA incC*$^{x-art}$ (DnaA-box#6/7) *dnaN cat recF* | | | pTR634 | pTR607 | BglII/FspAI | This work |
| pTR648 | *bla 'dnaA incC*$^{x-art}$ (DnaA-box#CR$^{44}$/6/7) *dnaN cat recF* | | | pTR634 | pTR608 | BglII/FspAI | This work |
| pTR653 | *bla 'dnaA incC*$^{art}$ (ΔGC-rich ΔDnaA-trios ΔAT-rich) *dnaN cat recF* | | | pTR634 | pTR614 | BglII/FspAI | This work |
| pTR664 | *bla 'dnaA incC*$^{x-art}$ (DnaA-box#Tm$^{45}$/6/7) *dnaN cat recF* | | | pTR634 | pOH18 | BglII/FspAI | This work |
| pTR699 | *bla amyE'* $P_{xyl}$-*dnaAchi*$^{R264A}$ *xylR spc 'amyE* | pHM610 | oTR1320/oTR1321 | | | | This work |
| pTR732 | *bla 'dnaA incC*$^{art}$ (DnaA-box#CR$^{49}$/6/7) *dnaN cat recF* | | | pHM327 | pTR587 | BglII/FspAI | This work |
| pTR733 | *bla 'dnaA incC*$^{art}$ (DnaA-box#CR$^{39}$/6/7) *dnaN cat recF* | | | pHM327 | pTR588 | BglII/FspAI | This work |

# Appendix Table 2. Plasmids

| | | | | | | | |
|---|---|---|---|---|---|---|---|
| pTR734 | *bla 'dnaA incC*$^{art}$ (DnaA-box#CR$^{33}$/6/7) *dnaN cat recF* | | | pHM327 | pTR589 | BglII/FspAI | This work |
| pTR736 | *bla 'dnaA incC*$^{art}$ (DnaA-box#CR$^{60}$/6/7) *dnaN'* | pTR424 | oTR1430/oTR1431 | | | | This work |
| pTR737 | *bla 'dnaA incC*$^{art}$ (DnaA-box#CR$^{65}$/6/7) *dnaN'* | pTR424 | oTR1432/oTR1433 | | | | This work |
| pTR738 | *bla 'dnaA incC*$^{art}$ (DnaA-box#CR$^{70}$/6/7) *dnaN'* | pTR424 | oTR1434/oTR1435 | | | | This work |
| pTR748 | *bla 'dnaA incC*$^{art}$ (DnaA-box#CR$^{60}$/6/7) *dnaN cat recF* | | | pHM327 | pTR736 | BglII/FspAI | This work |
| pTR749 | *bla 'dnaA incC*$^{art}$ (DnaA-box#CR$^{65}$/6/7) *dnaN cat recF* | | | pHM327 | pTR737 | BglII/FspAI | This work |
| pTR750 | *bla 'dnaA incC*$^{art}$ (DnaA-box#CR$^{70}$/6/7) *dnaN cat recF* | | | pHM327 | pTR738 | BglII/FspAI | This work |
| pTR834 | *bla 'dnaA incC* (ΔDnaA-box#3/4/5) *dnaN'* | pTR354 | oTR1575/oTR1576 | | | | This work |
| pTR835 | *bla 'dnaA incC* (ΔDnaA-box#2/4/5) *dnaN'* | pTR355 | oTR1576/oTR1577 | | | | This work |
| pTR836 | *bla 'dnaA incC* (ΔDnaA-box#3/4/5) *dnaN cat recF* | | | pHM327 | pTR834 | BglII/FspAI | This work |
| pTR837 | *bla 'dnaA incC* (ΔDnaA-box#2/4/5) *dnaN cat recF* | | | pHM327 | pTR835 | BglII/FspAI | This work |
| pTR864 | *bla 'dnaA incC* (ΔDnaA-box#4/5) *dnaN'* | pTR356 | oTR1576/oTR1605 | | | | This work |
| pTR865 | *bla 'dnaA incC* (ΔDnaA-box#4/5) *dnaN cat recF* | | | pHM327 | pTR864 | BglII/FspAI | This work |
| pTR881 | *bla amyE'* P$_{xyl}$-*dnaAchi*$^{R202A}$ *xylR spc 'amyE* | pHM610 | oTS24/oTS25 | | | | This work |
| pTR882 | *bla amyE'* P$_{xyl}$-*dnaAchi*$^{R206A}$ *xylR spc 'amyE* | pHM610 | oDS3/oDS4 | | | | This work |
| pTR883 | *bla amyE'* P$_{xyl}$-*dnaAchi*$^{F218A}$ *xylR spc 'amyE* | pHM610 | oDS47/oDS48 | | | | This work |
| pTR885 | *bla amyE'* P$_{xyl}$-*dnaAchi*$^{L269A}$ *xylR spc 'amyE* | pHM610 | oDS49/oDS50 | | | | This work |
| pTR887 | *bla amyE'* P$_{xyl}$-*dnaAchi*$^{R321A}$ *xylR spc 'amyE* | pHM610 | oDS51/oDS52 | | | | This work |
| pTR990 | *bla 'dnaA incC*$^{art}$ (DnaA-box#CR$^{86}$/6/7) *dnaN'* | pTR424 | oTR1801/oTR1824 | | | | This work |
| pTR991 | *bla 'dnaA incC*$^{art}$ (DnaA-box#CR$^{86}$/6/7) *dnaN cat recF* | | | pHM327 | pTR990 | BglII/FspAI | This work |
| pTR1009 | *bla 'dnaA incC*$^{art}$ (DnaA-box#CR$^{91}$/6/7) *dnaN'* | pTR424 | oTR1848/oTR1849 | | | | This work |
| pTR1010 | *bla 'dnaA incC*$^{art}$ (DnaA-box#CR$^{96}$/6/7) *dnaN'* | pTR424 | oTR1850/oTR1851 | | | | This work |
| pTR1012 | *bla 'dnaA incC*$^{art}$ (DnaA-box#CR$^{91}$/6/7) *dnaN cat recF* | | | pHM327 | pTR1009 | BglII/FspAI | This work |
| pTR1013 | *bla 'dnaA incC*$^{art}$ (DnaA-box#CR$^{96}$/6/7) *dnaN cat recF* | | | pHM327 | pTR1010 | BglII/FspAI | This work |
| pTR1043 | *bla 'dnaA incC*$^{art}$ (DnaA-box#CR$^{28}$/6/7) *dnaN'* | pTR424 | oTR1906/oTR1907 | | | | This work |
| pTR1044 | *bla 'dnaA incC*$^{art}$ (DnaA-box#CR$^{22}$/6/7) *dnaN'* | pTR424 | oTR1908/oTR1909 | | | | This work |
| pTR1045 | *bla 'dnaA incC*$^{art}$ (DnaA-box#CR$^{80}$/6/7) *dnaN'* | pTR424 | oTR1910/oTR1911 | | | | This work |
| pTR1046 | *bla 'dnaA incC*$^{art}$ (DnaA-box#CR$^{86}$/6/7) *dnaN'* | pTR424 | oTR1912/oTR1913 | | | | This work |
| pTR1047 | *bla 'dnaA incC*$^{art}$ (DnaA-box#CR$^{91}$/6/7) *dnaN'* | pTR424 | oTR1914/oTR1915 | | | | This work |
| pTR1051 | *bla 'dnaA incC*$^{art}$ (DnaA-box#CR$^{28}$/6/7) *dnaN cat recF* | | | pHM327 | pTR1043 | BglII/FspAI | This work |
| pTR1052 | *bla 'dnaA incC*$^{art}$ (DnaA-box#CR$^{22}$/6/7) *dnaN cat recF* | | | pHM327 | pTR1044 | BglII/FspAI | This work |
| pTR1053 | *bla 'dnaA incC*$^{art}$ (DnaA-box#CR$^{80}$/6/7) *dnaN cat recF* | | | pHM327 | pTR1045 | BglII/FspAI | This work |
| pTR1054 | *bla 'dnaA incC*$^{art}$ (DnaA-box#CR$^{86}$/6/7) *dnaN cat recF* | | | pHM327 | pTR1046 | BglII/FspAI | This work |
| pTR1055 | *bla 'dnaA incC*$^{art}$ (DnaA-box#CR$^{91}$/6/7) *dnaN cat recF* | | | pHM327 | pTR1047 | BglII/FspAI | This work |
| pUC18 | *bla lacZ'* DnaA-box(nt772-780) DnaA-box(nt866-874) | pUC18 contains two DnaA-boxes. DnaA-box(nt772-780) is equivalent to consensus DnaA-box#6 within *incC*. | | | | | Norrander et al. 1983 |
| pTS2 | *bla 'dnaA*$^{I190A}$ *incC dnaN cat recF* | pHM327 | oAK361/oAK362 | | | | This work |
| pTS4 | *bla 'dnaA*$^{K222A}$ *incC dnaN cat recF* | pHM327 | oAK365/oAK366 | | | | This work |
| pTS5 | *bla 'dnaA*$^{Q224A}$ *incC dnaN cat recF* | pHM327 | oAK367/oAK368 | | | | This work |
| pTS6 | *bla 'dnaA*$^{T225A}$ *incC dnaN cat recF* | pHM327 | oAK373/oAK374 | | | | This work |
| pTS7 | *bla 'dnaA*$^{R264A}$ *incC dnaN cat recF* | pHM327 | oAK369/oAK370 | | | | This work |
| pTS10 | *bla 'dnaA*$^{N187A}$ *incC dnaN cat recF* | pHM327 | oTS4/oTS5 | | | | This work |
| pTS15 | *bla 'dnaA*$^{R202A}$ *incC dnaN cat recF* | pHM327 | oTS24/oTS25 | | | | This work |

# Appendix Table 3. Quickchange oligonucleotides

| Product | Template | Primer #1 | Sequence (5'→3') | Primer #2 | Sequence (5'→3') |
|---|---|---|---|---|---|
| pCB3 | pHM327 | oCB5 | GAATTTTTCGCTACATTTAACACATTACACGAAG | oCB6 | GTTAAATGTAGCGAAAAATTCTTCCTGGGTTTG |
| pCB6 | pHM327 | oCB11 | GAACAAGCATTAATCAGAGTTGTCGCTTATTC | oCB12 | GATTAATGCTTGTTCGAGTTCCCGAATATTGC |
| pDS3 | pHM327 | oDS3 | CTATGCTAATGTTGATGTGCTTTTGATAG | oDS4 | CATTAGCATAGCGATTGCGGAAGTCGAC |
| pDS12 | pHM327 | oDS7 | CTTCTGCTAAATTTACAAACGAATTCATC | oDS8 | GTAAATTTAGCAGAAGACAGATAAACCACTTTG |
| pDS52 | pHM327 | oDS45 | CGAGCTGCACATGCTGCTTCCCTCGCAG | oDS46 | GTGCAGCTCGGTTTCCAGATCCGATGAC |
| pDS53 | pHM327 | oDS47 | CAAGCTTTAGCGGGGAAAGAACAAACC | oDS48 | CTAAAGCTTGAATATCATCTATCAAAAG |
| pDS54 | pHM327 | oDS51 | CATTAATCGCTGTTGTCGCTTATTCATCTTTAATTAATAAAG | oDS52 | CAACAGCGATTAATGCTCCTTCGAGTTC |
| pDS65 | pHM327 | oDS49 | GGAGCTATTACAGATATCACACCGCCTG | oDS50 | GTAATAGCTCCCCATTCAAAACGTGAGC |
| pOH18 | pTR424 | oOH28 | TAAACCTACCACCGCGGTATCAGCTCACTCAAAG | oOH29 | CGGTGGTAGGTTTAACGACCGAGCGCAGCGAG |
| pTR27 | pHM367 | oTR118 | GAAAGGCAAGGAAGCTTTTCGGAAGTCATACACAGTCTGTC | oTR119 | AAAGCTTCCTTGCCTTTCCCCGATTGATCCCCGGTCCTG |
| pTR29 | pHM367 | oTR82 | CACAGTCTTCCTTGCTGTGGATAGGCTGTGTTTCCTGTCTTTTTC | oTR83 | ATCCACAGCAAGGAAGACTGTGTATGACTTCCGAAAAGTTATTC |
| pTR33 | pHM367 | oTR90 | CCACATTCCTTGCGGCCCTACTATTACTTCTAC | oTR91 | GGGCCGCAAGGAATGTGGATAAGTTGTGAAAAAGACAGGAAAC |
| pTR35 | pTR42 | oTR102 | ACATATTTTTTATAAATATATATATTAATAGGAACCGTAAAAAGGCCGCGTTGCTG | oTR103 | CCTATTAATATATATATTTATAAAAAATATGTTCTTTCCTGCGTTATCCCCTGATTC |
| pTR42 | pUC18 | oTR96 | GGCGTTGTTCCATAGGCTCCGCCCCCCTGACGAGCATC | oTR97 | TGGAACAACGCCAGCAACGCGGCCTTTTTACGG |
| pTR60 | pTR33 | oTR134 | CTGTCCTTCCTTGCACTTATCCACATTCCTTGCGGCCCTAC | oTR135 | TAAGTGCAAGGAAGGACAGGAAACACAGCCTATCCACATGTGGACAG |
| pTR61 | pHM367 | oTR136 | CACAACCTTCCTTGCTTCCTTGCGGCCCTACTATTACTTC | oTR137 | GGAAGCAAGGAAGGTTGTGAAAAAGACAGGAAACACAGCCTATCC |
| pTR114 | pTR35 | oTR334 | GGCCCTACTATTACTTCTACTATTTTTTATAAATATATATATTAATAGGAACCGTAAAAAG | oTR335 | GTAGAAGTAATAGTAGGGCCCCTGATTCTGTGGATAAC |
| pTR116 | pHM367 | oTR217 | CAACTGTGGATAAAATCCACAGGCCCTACTATTACTTCTAC | oTR218 | GATTTTATCCACAGTTGTGAAAAAGACAGGAAACACAG |
| pTR117 | pTR116 | oTR219 | TTTGTGGATAAGGCCCTACTATTACTTCTAC | oTR220 | CCTTATCCACAAATCCACAGTTGTGAAAAAGAC |
| pTR120 | pTR27 | oTR227 | GAAGGATATAGTTGTCTGTCCACATGTGGATAGGCTGTGTTTCC | oTR228 | AGACAACTATATCCTTCCGAAAAGCTTCCTTGCCTTTCC |
| pTR121 | pTR120 | oTR229 | TGTCTTCCTTGCTGTGGATAGGCTGTGTTTCCTGTCTTTTTC | oTR230 | ACAGCAAGGAAGACAACTATATCCTTCCGAAAAGCTTC |
| pTR122 | pTR121 | oTR231 | TTGCAGATATCGTGCTGTGTTTCCTGTCTTTTTCACAAC | oTR232 | CAGCACGATATCTGCAAGGAAGACAACTATATCCTTCCGAAAAG |
| pTR123 | pTR122 | oTR233 | TGTCCTTCCTTGCACTTATCCACAAATCCACAGGCCCTAC | oTR234 | AAGTGCAAGGAAGGACAGGAAACACAGCACGATATCTGCAAG |
| pTR155 | pTR123 | oTR90 | CCACATTCCTTGCGGCCCTACTATTACTTCTAC | oTR153 | GGCCGCAAGGAATGTGGATAAGTGCAAGGAAGGACAGGAAAC |
| pTR198 | pTR155 | oTR366 | GTCTTTTTCACAACTTATCCACATTCCTTGCGGCCCTAC | oTR367 | AGTTGTGAAAAAGACAGGAAACACAGCACGATATCTGCAAG |
| pTR204 | pTR114 | oTR374 | ACAAATCCACAGGCCCTACTATTACTTCTAC | oTR375 | CCTGTGGATTTGTGGATAACCGTATTACCGCCTTTG |
| pTR213 | pHM367 | oTR393 | CGTATATATCACTACGATACGGGAGGGCTTACCATCTG | oTR394 | AGTGATATATACGACGGGGAGTCAGGCAACTATGGATG |
| pTR214 | pTR213 | oTR404 | AATACGGGAATCAGGGGATAACGCAGGAAAG | oTR405 | CTGATTCCCGTATTACCGCCTTTGAGTGAG |
| pTR307 | pTR60 | oTR118 | GAAAGGCAAGGAAGCTTTTCGGAAGTCATACACAGTCTGTC | oTR119 | AAAGCTTCCTTGCCTTTCCCCGATTGATCCCCGGTCCTG |
| pTR322 | pTR307 | oTR483 | CAAGATATCGTGCTGTGTTTCCTGTCCTTCCTTG | oTR484 | GCACGATATCTTGTGGACAGACTGTGTATGACTTC |
| pTR327 | pTR29 | oTR488 | AAGGATATAGTTGTCTTCCTTGCTGTGGATAGGCTGTGTTTC | oTR489 | ACAACTATATCCTTCCGAAAAGTTATTCACACTTTCC |
| pTR354 | pTR123 | oTR519 | GTCATACACAGTCTTCCTTGCAGATATCGTGCTGTGTTTC | oTR520 | GACTGTGTATGACTTCCGAAAAGCTTCCTTGCCTTTC |
| pTR355 | pTR123 | oTR521 | GTCTGTCCACAAGATATCGTGCTGTGTTTCCTGTC | oTR522 | CTTGTGGACAGACAACTATATCCTTCCGAAAAGCTTC |
| pTR356 | pTR322 | oTR523 | CAAATCCACAGGCCCTACTATTACTTCTACTATTTTTTATAAATATATATATTAATAC | oTR524 | CCTGTGGATTTGTGGATAAGTGCAAGGAAGGAC |
| pTR365 | pTR327 | oTR231 | TTGCAGATATCGTGCTGTGTTTCCTGTCTTTTTCACAAC | oTR232 | CAGCACGATATCTGCAAGGAAGACAACTATATCCTTCCGAAAAG |
| pTR392 | pTR365 | oTR233 | TGTCCTTCCTTGCACTTATCCACAAATCCACAGGCCCTAC | oTR234 | AAGTGCAAGGAAGGACAGGAAACACAGCACGATATCTGCAAG |
| pTR424 | pTR228 | oTR604 | CCTGATTCAACCGCCTCTCCCCGC | oTR605 | AGGCGGTTGAATCAGGGGATAACGCAGGAAAG |
| pTR504 | pHM610 | oTR415 | GAATTCGCCAACTCTATCCGAGATAATAAAGCCGTC | oTR416 | GAGTTGGCGAATTCGTTTGTAAATTTCTCAGAAGAC |
| pTR548 | pTR424 | oTR1062 | TCCTTATCCACAGACTCGCTGCGCTCGGTC | oTR1063 | GTCTGTGGATAAGGAAGCGGAAGAGCGCCCAATAC |
| pTR550 | pTR424 | oTR1066 | CGTTATCCACATCGGCTGCGGCGAGCG | oTR1067 | CGATGTGGATAACGCAGCGAGTCAGTGAGCGAGGAAG |
| pTR552 | pTR424 | oOH51 | GTTCTTATCCACAGAGCGGTATCAGCTCACTCAAAGG | oOH52 | CTCTGTGGATAAGAACGACCGAGCGCAGCGAGTCAGTG |
| pTR587 | pTR424 | oTR1177 | GGTTTATCCACAGCGGCGAGCGGTATCAG | oTR1178 | GCTGTGGATAAACCGAGCGCAGCGAGTC |
| pTR588 | pTR424 | oTR1179 | CTGTTATCCACAGTATCAGCTCACTCAAAGGCGGTAATAC | oTR1180 | ATACTGTGGATAACAGCCGAACGACCGAGCGCAGCGAGTC |
| pTR589 | pTR424 | oTR1181 | CGATTATCCACAGCTCACTCAAAGGCGGTAATAC | oTR1182 | GCTGTGGATAATCGCCGCAGCCGAAC |
| pTR595 | pTR607 | oTR1189 | AGGTTATCCACACGCATCAGGCGCCATTCG | oTR1190 | CGTGTGGATAACCTTACGCATCTGTGCGGTATTTC |
| pTR598 | pTR607 | oTR1195 | GAGTTATCCACAGCTTGTCTGTAAGCGGATG | oTR1196 | GCTGTGGATAACTCCGGGAGCTGCATGTG |
| pTR600 | pTR607 | oTR1199 | AAATTATCCACAGTTCCGCGCACATTTCCCC | oTR1200 | AACTGTGGATAATTTATTTTTCTAAATACACTATTTAAGCTGTTCTTTAATTTC |
| pTR606 | pTR552 | oTR1201 | ATATTTGAATCAGGCTGCGCAACTGTTG | oTR1202 | AGCCTGATTCAAATATGTATCCGCTCATGAGACAATAAC |
| pTR607 | pTR608 | oTR1221 | GGCTGCGGCGAGCGGTATCAGCTCACTCAAAG | oTR1222 | GCCGCAGCCGAACGACCGAGCGCAGCGAGTC |
| pTR610 | pTR615 | oTR1209 | GGCTTCCTTGCAATCCACAGGCCCTACTATTACTTCTAC | oTR1210 | TTGCAAGGAAGCCGTATTACCGCCTTTGAGTGAG |
| pTR611 | pTR610 | oTR1211 | GCGAATCAGGGGCCCTACTATTACTTCTACTATTTTTTATAAATATATATATTAATAC | oTR1212 | CCCCTGATTCGCAAGGAAGCCGTATTACCG |
| pTR612 | pTR611 | oTR1213 | TCAGGGGATATACTATTACTTCTACTATTTTTTATAAATATATATATTAATACATTATC | oTR1214 | TAGTATATCCCCTGATTCGCAAGGAAG |
| pTR613 | pTR612 | oTR1215 | GCAGGAAAGAACATGTTTTTTATAAATATATATATTAATACATTATCCGTTAGGAG | oTR1216 | ATGTTCTTTCCTGCGTTATCCCCTGATTCGCAAGGAAG |
| pTR614 | pTR613 | oTR1217 | AAAGGCCAGCAAAAGGCCACATTATCCGTTAGGAGGATAAAAATGAAATTC | oTR1218 | TTTGCTGGCCTTTTGCTCACATGTTCTTTCCTGCGTTATCCC |
| pTR615 | pTR424 | oTR1219 | AGACAGATCTTTTCCATAGGCTCCGCCCCCCTGAC | oTR1220 | TGGAAAAGATCTGTCTTCTGGAATATCAAGTTTCG |
| pTR621 | pHM610 | oTR368 | GGGGGCAGAACAAACCCAGGAAGAATTTTTC | oTR369 | TTCTGCCCCCGCTAAAAATTGAATATCATCTATC |
| pTR622 | pHM610 | oTR372 | ACAAGCCCAGGAAGAATTTTTCCATACATTTAAC | oTR373 | TGGGCTTGTTCTTTCCCCGCTAAAAATTG |
| pTR634 | pHM327 | oTR1251 | GACTGGCGTAAAAAGGCCGCGTTGCTG | oTR1252 | TTTTTACGCCAGTCATTAGGCCTTTGCTGTTTG |
| pTR699 | pHM610 | oTR1320 | CGCTCAGCTTTTGAATGGGGACTTATTACAGATATCAC | oTR1321 | TCAAAAGCTGAGCGCAATCTGTCTTCAAGTGTC |
| pTR736 | pTR424 | oTR1430 | TCGTTATCCACAGTCGTTCGGCTGCGGCGAG | oTR1431 | GACTGTGGATAACGAGTCAGTGAGCGAGGAAG |

# Appendix Table 3. Quickchange oligonucleotides

| | | | | | |
|---|---|---|---|---|---|
| pTR737 | pTR424 | oTR1432 | CTGTTATCCACAGCTCGGTCGTTCGGCTG | oTR1433 | AGCTGTGGATAACAGTGAGCGAGGAAGCGGAAG |
| pTR738 | pTR424 | oTR1434 | GCTTTATCCACAGCTGCGCTCGGTCGTTC | oTR1435 | AGCTGTGGATAAAGCGAGGAAGCGGAAGAGC |
| pTR834 | pTR354 | oTR1575 | AAAGTGTGAATAACTTTTCGGAAGTCATACACAGTCTTC | oTR1576 | AAGTTATTCACACTTTCCCCGATTGATCCCCGGTC |
| pTR835 | pTR355 | oTR1577 | AAAGTGTGAATAACTTTTCGGAAGGATATAGTTGTCTGTC | oTR1576 | AAGTTATTCACACTTTCCCCGATTGATCCCCGGTC |
| pTR864 | pTR356 | oTR1605 | AAGTGTGAATAACTTTTCGGAAGTCATACACAGTC | oTR1576 | AAGTTATTCACACTTTCCCCGATTGATCCCCGGTC |
| pTR881 | pHM610 | oTS24 | CTTCGCAAATCGCTATCGAAATGTTGATG | oTS25 | GATTTGCGAAGTCGACGGCTTTATTATC |
| pTR882 | pHM610 | oDS3 | CTATGCTAATGTTGATGTGCTTTTGATAG | oDS4 | CATTAGCATAGCGATTGCGGAAGTCGAC |
| pTR883 | pHM610 | oDS47 | CAAGCTTTAGCGGGGAAAGAACAAACC | oDS48 | CTAAAGCTTGAATATCATCTATCAAAAG |
| pTR885 | pHM610 | oDS49 | GGAGCTATTACAGATATCACACCGCCTG | oDS50 | GTAATAGCTCCCCATTCAAAACGTGAGC |
| pTR887 | pHM610 | oDS51 | CATTAATCGCTGTTGTCGCTTATTCATCTTTAATTAATAAAG | oDS52 | CAACAGCGATTAATGCTCCTTCGAGTTC |
| pTR990 | pTR424 | oTR1801 | GGTTATCCACAAGCGCCCAATACGCACTATTTAAG | oTR1824 | GCTTGTGGATAACCTCGCTCACTGACTCGC |
| pTR1009 | pTR424 | oTR1848 | GGTGTGGATAACGCTTCCTCGCTCACTGAC | oTR1849 | CGTTATCCACACCAATACGCACTATTTAAGCTG |
| pTR1010 | pTR424 | oTR1850 | CGTTGTGGATAATCTTCCGCTTCCTCGCTCAC | oTR1851 | AGATTATCCACAACGCACTATTTAAGCTGTTCTTTAATTTC |
| pTR1043 | pTR424 | oTR1906 | GGTTTATCCACACTCAAAGGCGGTAATACGGTTATC | oTR1907 | GAGTGTGGATAAACCGCTCGCCGCAGC |
| pTR1044 | pTR424 | oTR1908 | AGCTTATCCACAGGCGGTAATACGGTTATCCAC | oTR1909 | CCTGTGGATAAGCTGATACCGCTCGCC |
| pTR1045 | pTR424 | oTR1910 | CCGTTATCCACATCACTGACTCGCTGCGCTCGGTC | oTR1911 | GATGTGGATAACGGAAGAGCGCCCAATACG |
| pTR1046 | pTR424 | oTR1912 | GCTTTATCCACACCTCGCTCACTGACTCGC | oTR1913 | GGTGTGGATAAAGCGCCCAATACGCACTATTTAAG |
| pTR1047 | pTR424 | oTR1914 | TGGTTATCCACACGCTTCCTCGCTCACTGAC | oTR1915 | CGTGTGGATAACCAATACGCACTATTTAAGCTGTTCTTTAATTTC |
| pTR1048 | pTR424 | oTR1916 | CGTTATCCACACTCTTCCGCTTCCTCGCTCAC | oTR1917 | GAGTGTGGATAACGCACTATTTAAGCTGTTCTTTAATTTCTTTTAC |
| pTS2 | pHM327 | oAK361 | GAATTCGCTAACTCTATCCGAGATAATAAAGCCGTC | oAK362 | GAGTTAGCGAATTCGTTTGTAAATTTCTCAGAAGAC |
| pTS4 | pHM327 | oAK365 | CGGGGGCTGAACAAACCCAGGAAGAATTTTTCCATAC | oAK366 | GTTCAGCCCCCGCTAAAAATTGAATATCATCTATC |
| pTS5 | pHM327 | oAK367 | AAGAAGCTACCCAGGAAGAATTTTTCCATAC | oAK368 | GGGTAGCTTCTTTCCCCGCTAAAAATTGAATATC |
| pTS6 | pHM327 | oAK373 | GAACAAGCTCAGGAAGAATTTTTCCATACATTTAAC | oAK374 | CCTGAGCTTGTTCTTTCCCCGCTAAAAATTGAATATC |
| pTS7 | pHM327 | oAK369 | GCTCAGCTTTTGAATGGGGACTTATTACAGATATC | oAK370 | TCAAAAGCTGAGCGCAATCTGTCTTCAAGTGTCGG |
| pTS10 | pHM327 | oTS4 | GAAATTTACAGCAGAATTCATCAACTCTATCCGAG | oTS5 | GAATTCTGCTGTAAATTTCTCAGAAGACAG |
| pTS15 | pHM327 | oTS24 | CTTCGCAAATCGCTATCGAAATGTTGATG | oTS25 | GATTTGCGAAGTCGACGGCTTTATTATC |

# Appendix Table 4. DNA scaffolds

| Name | | 5′→3′ (DNA scaffold) | | 5′→3′ (DNA scaffold) | | 5′→3′ (DNA scaffold) | | 5′→3′ (competitor) | Figure |
|---|---|---|---|---|---|---|---|---|---|
| Wild-type | oTR602 | ACTTATCCACAAATCCACAGGCCC | oHM590 | Cy5-TACTATTACTTCTACTA | oHM558 | TAGTAGAAGTAATAGTAGGGCCTGTGGATTTGTGGATAAGT | oTR964 | TACTATTACTTCTACTA | 5C,6B,7C, S5 |
| Wild-type | oTR602 | ACTTATCCACAAATCCACAGGCCC | oTR964 | TACTATTACTTCTACTA | oHM558 | TAGTAGAAGTAATAGTAGGGCCTGTGGATTTGTGGATAAGT | | | 5E,6F |
| Wild-type | oHM752 | Cy3-ACTTATCCACAAATCCACAGGCCC | oHM590 | Cy5-TACTATTACTTCTACTA | oHM558 | TAGTAGAAGTAATAGTAGGGCCTGTGGATTTGTGGATAAGT | | | 5G |
| ΔDnaA-boxes | oHM602 | ACCTTCCTTGCTTCCTTGCGGCCC | oHM590 | Cy5-TACTATTACTTCTACTA | oHM601 | TAGTAGAAGTAATAGTAGGGCCGCAAGGAAGCAAGGAAGGT | oTR964 | TACTATTACTTCTACTA | 6B,7C |
| ΔDnaA-trios | oTR602 | ACTTATCCACAAATCCACAGGCCC | oHM599 | Cy5-ATGATAATGAAGATGAT | oHM598 | ATCATCTTCATTATCATGGGCCTGTGGATTTGTGGATAAGT | oHM600 | ATGATAATGAAGATGAT | 6B,7C |
| Wild-type | oHM616 | CGTTCGGCTGCGGCGAGCGGTATCAGCTCACTCAAAG GCGGTAATACGGTTATCCACAAATCCACAGGCCC | oHM590 | Cy5-TACTATTACTTCTACTA | oHM615 | TAGTAGAAGTAATAGTAGGGCCTGTGGATTTGTGGATAACCGTATTACCGCCT TTGAGTGAGCTGATACCGCTCGCCGCAGCCGAACG | | | 6D,7E |
| ΔDnaA-boxes | oHM753 | CGTTCGGCTGCGGCGAGCGGTATCAGCTCACTCAAAG GCGGTAATACGGCTTCCTTGCTTCCTTGCGGCCC | oHM590 | Cy5-TACTATTACTTCTACTA | oHM754 | TAGTAGAAGTAATAGTAGGGCCGCAAGGAAGCAAGGAAGCCGTATTACCGCCT TTGAGTGAGCTGATACCGCTCGCCGCAGCCGAACG | | | 6D |
| ΔDnaA-trios | oHM616 | CGTTCGGCTGCGGCGAGCGGTATCAGCTCACTCAAAG GCGGTAATACGGTTATCCACAAATCCACAGGCCC | oHM599 | Cy5-ATGATAATGAAGATGAT | oHM755 | ATCATCTTCATTATCATGGGCCTGTGGATTTGTGGATAACCGTATTACCGCCT TTGAGTGAGCTGATACCGCTCGCCGCAGCCGAACG | | | 6D |
| ΔDnaA-trios | oTR602 | ACTTATCCACAAATCCACAGGCCC | oHM600 | ATGATAATGAAGATGAT | oHM598 | ATCATCTTCATTATCATGGGCCTGTGGATTTGTGGATAAGT | | | 6F |
| Wild-type | oTR602 | ACTTATCCACAAATCCACAGGCCC | oHM576 | Cy5-TACTACTACTACTACTA | oHM757 | TAGTAGTAGTAGTAGTAGGGCCTGTGGATTTGTGGATAAGT | oHM758 | TACTACTACTACTACTA | 6H |
| ΔA | oTR602 | ACTTATCCACAAATCCACAGGCCC | oHM759 | Cy5-ACTACTACTACTACTAT | oHM760 | ATAGTAGTAGTAGTAGTGGGCCTGTGGATTTGTGGATAAGT | oHM761 | ACTACTACTACTACTAT | 6H |
| ΔATG | oTR602 | ACTTATCCACAAATCCACAGGCCC | oHM764 | Cy5-TACTACTACTACTATAC | oHM765 | GTATAGTAGTAGTAGTAGGGCCTGTGGATTTGTGGATAAGT | oHM766 | TACTACTACTACTATAC | 6H |
| Wild-type | oSP330 | Biotin- CGTTCGGCTGCGGCGAGCGGTATCAGCTCACTCAAAG GCGGTAATACGGTTATCCACAAATCCACAGGCCC | oHM590 | Cy5-TACTATTACTTCTACTA | oHM615 | TAGTAGAAGTAATAGTAGGGCCTGTGGATTTGTGGATAACCGTATTACCGCCT TTGAGTGAGCTGATACCGCTCGCCGCAGCCGAACG | oTR964 | TACTATTACTTCTACTA | 7E,S6 |
