# Supplementary material for: Identification of a basal system for unwinding a bacterial chromosome origin
Source: EMBO J. 2019 Jun 27;38(15):e101649. doi: 10.15252/embj.2019101649 (PMC6669920; doi:10.15252/embj.2019101649)
Supplement: Supplementary file 2 — Expanded View Figures PDF [file EMBJ-38-e101649-s002.pdf]

Expanded View Figures

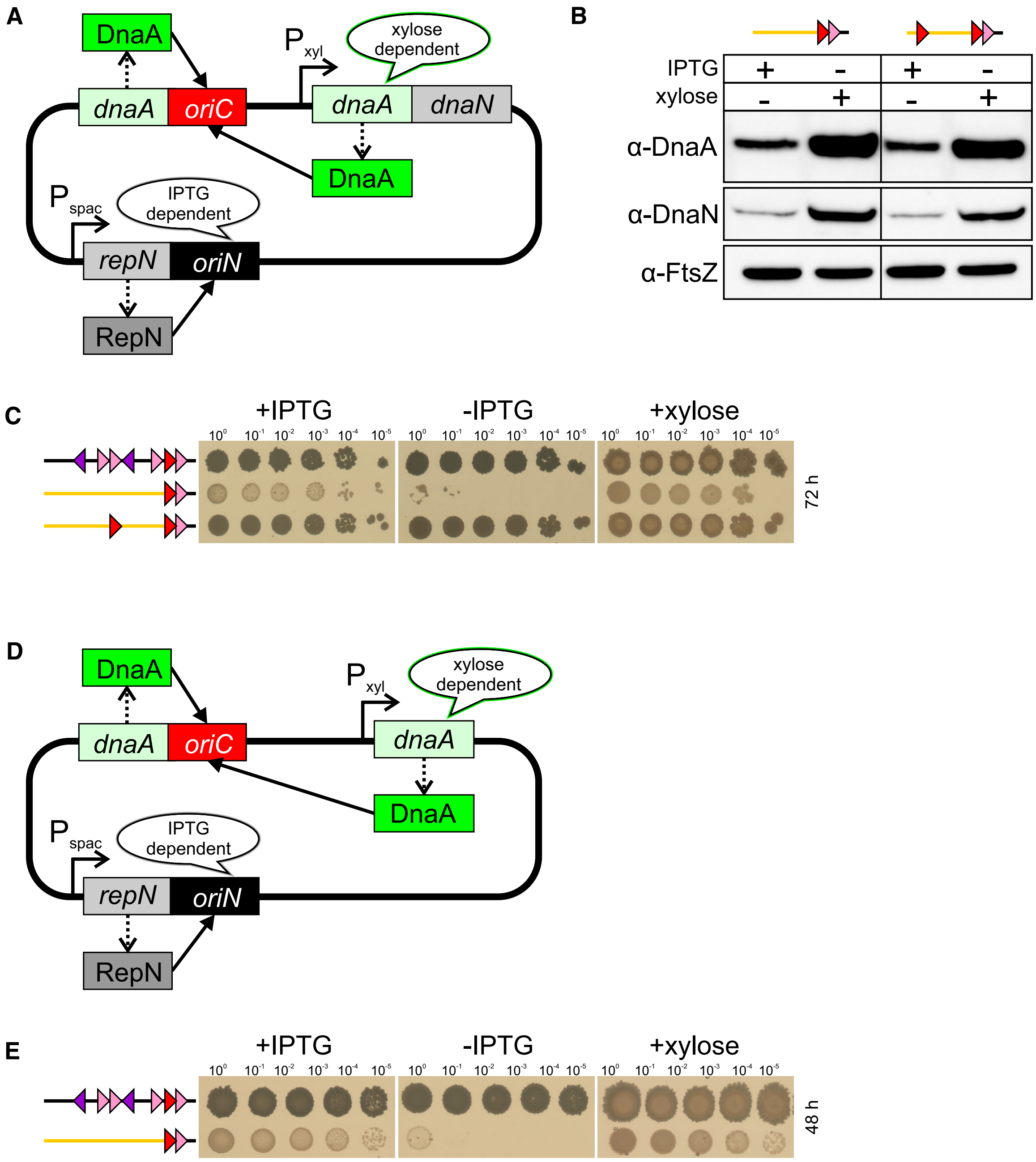

Figure EV1. Overexpression of DnaA identifies the minimal sequences of the origin unwinding region.

B Immunoblot of DnaA and DnaN with and without induction of the ectopic *dnaA-dnaN* operon with xylose. The tubulin homolog FtsZ was used as a loading control. *incC*^{art} DnaA-box#6/7 (TR684), *incC*^{art} DnaA-box# CR^{44}/6/7 (TR698).

C Overexpression of DnaA and DnaN suppresses deletion of the distal DnaA-box. *incC* (TR672), *incC*^{art} DnaA-box#6/7 (TR684), *incC*^{art} DnaA-box# CR^{44}/6/7 (TR698).

D The *oriC*-independent strain used for overexpressing DnaA.

E Overexpression of DnaA suppresses deletion of the distal DnaA-box. *incC* (TR671), *incC*^{art} DnaA-box#6/7 (TR674).

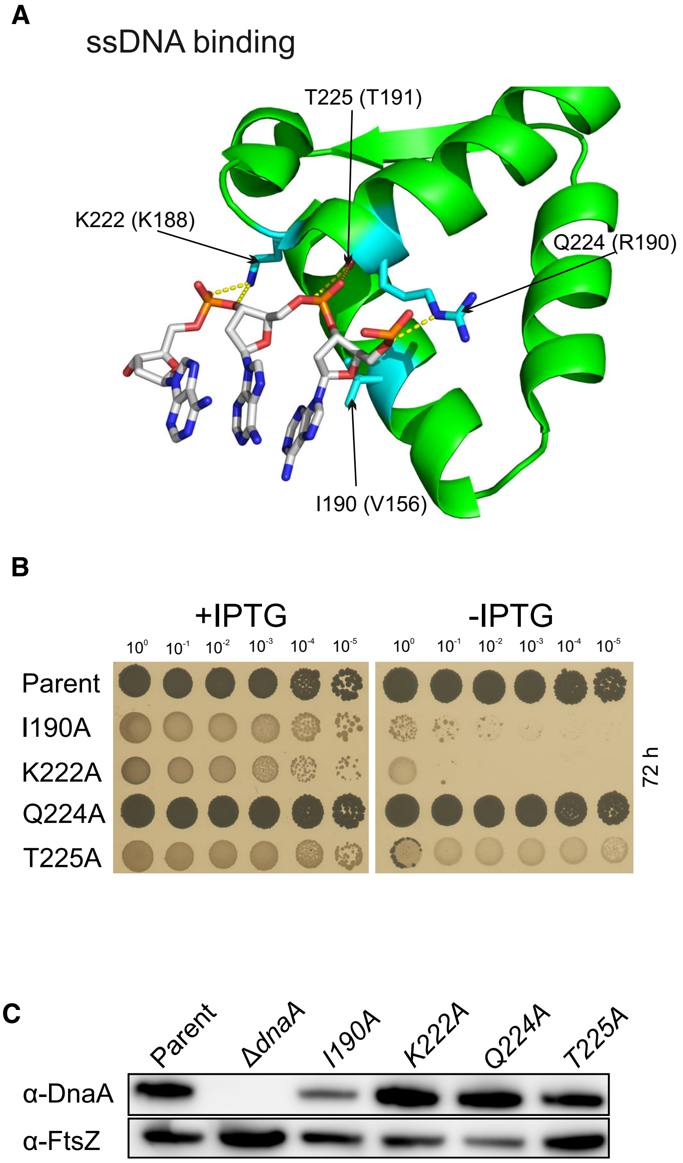

Figure EV2. Essentiality of proposed DnaA ssDNA binding residues in *B. subtilis*.

A DnaA residues implicated in binding ssDNA mapped onto the crystal structure from *A. aeolicus* (PDB ID 1L8Q). Residues are labelled according to the *B. subtilis* numbering, with *A. aeolicus* shown in parentheses.

B Analysis of *B. subtilis* DnaA variants using the inducible *oriN* strain. Parent (HM1108), $DnaA^{I190A}$ (DS18), $DnaA^{K222A}$ (DS53), $DnaA^{Q224A}$ (DS54), $DnaA^{T225A}$ (DS52).

C Immunoblot analysis of the DnaA variants. The tubulin homolog FtsZ was used as a loading control. Strains are the same as panel B, with the addition of Δ*dnaA* (HM1423).

 © 2019 The Authors

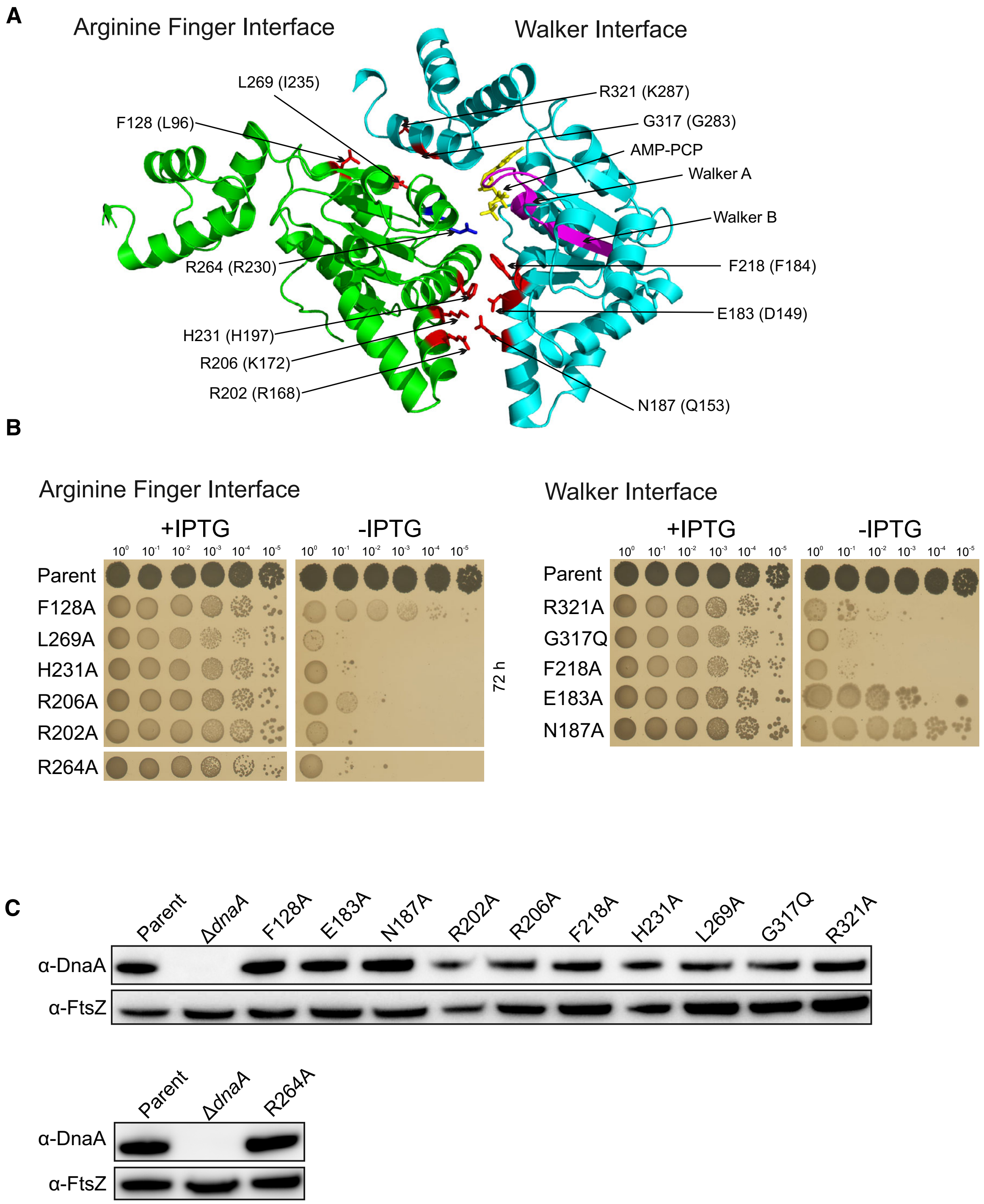

Figure EV3. Essentiality of proposed DnaA filament formation residues in *B. subtilis*.

A DnaA residues implicated in the AAA+/AAA+ filament formation interface mapped onto the crystal structure from *A. aeolicus* (PDB ID 2HCB). Residues are labelled according to the *B. subtilis* numbering, with *A. aeolicus* shown in parentheses.

B Analysis of *B. subtilis* DnaA variants using the inducible *oriN* strain. Residues have been divided according to the respective AAA+ interface. Parent (HM1108), $DnaA^{F128A}$ (DS25), $DnaA^{L269A}$ (DS34), $DnaA^{H231A}$ (DS50), $DnaA^{R206A}$ (DS22), $DnaA^{R202A}$ (DS21), $DnaA^{R264A}$ (DS56), $DnaA^{R321A}$ (DS27), $DnaA^{G317Q}$ (DS51), $DnaA^{F218A}$ (DS26), $DnaA^{E183A}$ (DS6), $DnaA^{N187A}$ (DS23).

C Immunoblot analysis of the DnaA variants. The tubulin homolog FtsZ was used as a loading control. Strains are the same as panel B, with the addition of Δ*dnaA* (HM1423).

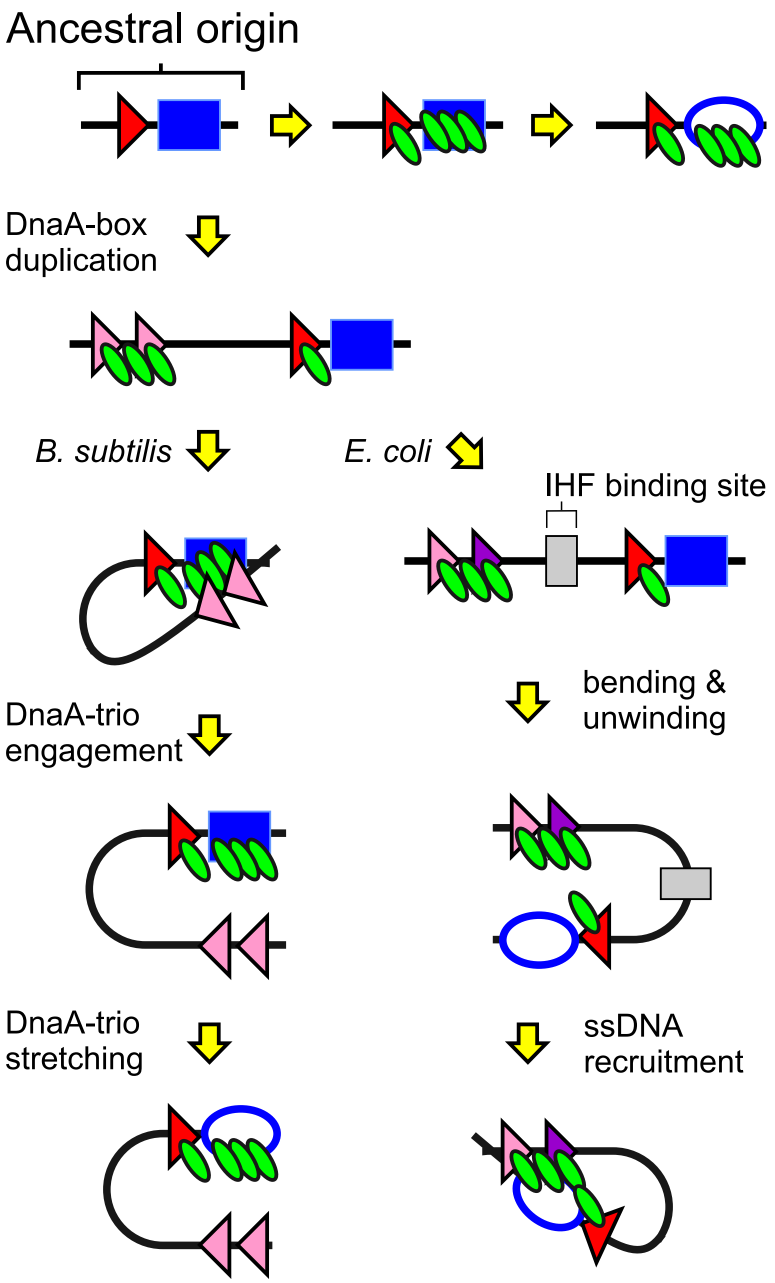

Figure EV4. Model for bacterial origin diversification.
Starting with the ancestral origin architecture of a DnaA-box (triangle) adjacent to the unwinding site (blue box), additional DnaA-boxes could arise through duplication and/or mutation. In *B. subtilis*, these upstream DnaA-boxes create a robust mechanism that increases the local concentration of DnaA (green) at the unwinding site. In *E. coli*, the origin appears to have undergone further modification by varying the affinity of upstream DnaA-boxes towards DnaA and with the addition of a specific site for the nucleoid-associated protein IHF (grey) (Leonard & Grimwade, 2015). It has been proposed that DNA bending by DnaA and IHF induces topological strain that promotes strand separation, after which a DNA loop delivers ssDNA at the unwinding site to an upstream DnaA oligomer (Sakiyama *et al*, 2017; Grimwade *et al*, 2018).

© 2019 The Authors

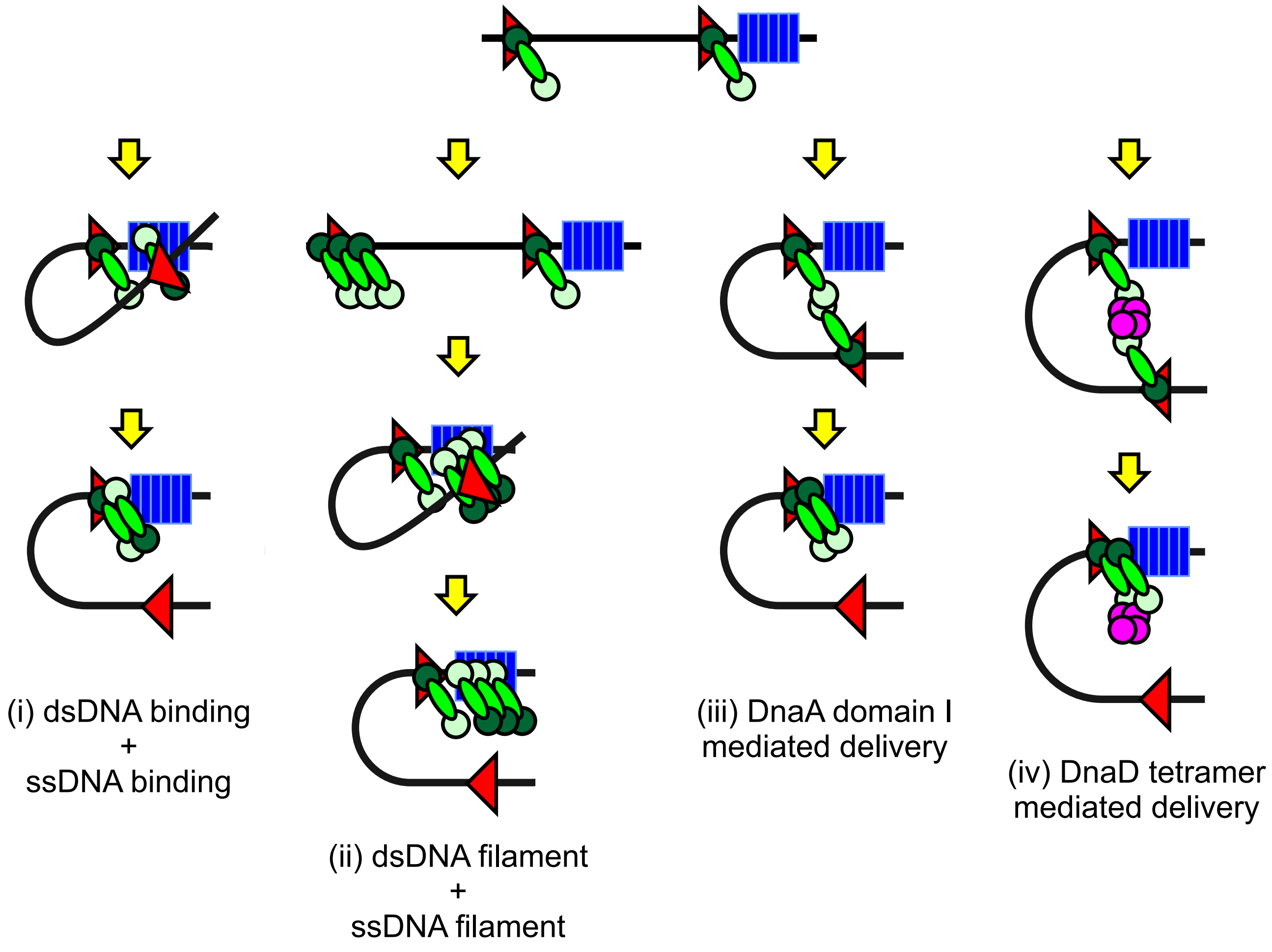

Figure EV5. Models for DnaA looping at *B. subtilis incC*.
There are multiple possibilities for the architecture and composition of the proposed DNA loop at *incC* that delivers DnaA (green) to the DnaA-trios (blue). For clarity only, two DnaA-boxes (red) are shown. (i) A DNA loop with DnaA monomers bound at each DnaA-box. Here, the DnaA protein being delivered would contact both dsDNA and ssDNA. (ii) A DNA loop involving the delivery of a DnaA filament. Here, the DnaA filament being delivered would contact both dsDNA and ssDNA. (iii) A DNA loop facilitated by a homodimer intermediate of DnaA mediated by domain I. (iv) A DNA loop promoted by an oligomeric scaffold protein (magenta).

© 2019 The Authors
